# *Rehmannia glutinosa DC.-Lilium lancifolium Thunb.* in the treatment of depression: a comprehensive review and perspectives

**DOI:** 10.3389/fphar.2024.1471307

**Published:** 2024-10-30

**Authors:** ZongHao Wang, Xiaoyu Wang, Xiangyu Mou, ChangLin Wang, Ya Sun, JieQiong Wang

**Affiliations:** ^1^ The College of Pharmacy Shandong University of Traditional Chinese Medicine, Jinan, Shandong, China; ^2^ The College of Chinese Medicine is Part of the Shandong University of Traditional Chinese Medicine in Jinan, Jinan, Shandong, China; ^3^ Research Institute for Traditional Chinese Medicine, Shandong University of Traditional Chinese Medicine, Jinan, Shandong, China

**Keywords:** *Lilium*, *Rehmannia glutinosa*, depression, active metabolites, pharmacological mechanism

## Abstract

**Background:**

In recent years, the incidence of depression, recognized as a serious psychological disorder, has escalated rapidly. *Rehmannia glutinosa DC.* (Scrophulariaceae; Rehmanniae Radix, Crude drug) and *Lilium lancifolium Thunb.* (Liliaceae; Lilii bulbus, Crude drug) constitute a classic anti-depressant combination, exhibiting pharmacological effects that include anti-depressive, anti-anxiety, and anti-inflammatory properties. Current clinical studies have demonstrated that Baihe Dihuang Decoction, a traditional Chinese herbal compound, is effective in treating depression. However, the majority of scholars have predominantly examined *Rehmannia glutinos*a and *Lilium* in isolation, and a comprehensive elucidation of their principal active metabolites and pharmacological mechanisms remains lacking.

**Methods:**

A comprehensive literature search was conducted as of 29 September 2024, utilizing databases such as PubMed, CNKI, Wanfang Data, Baidu Scholar, and Google Scholar. Additionally, classical texts on Chinese herbal medicine, the Chinese Pharmacopoeia, as well as doctoral and master’s theses, were included in the collected materials. The search employed specific terms including “*R. glutinosa*,” “*Lilium*,” “Baihe Dihuang decoction,” “application of Baihe Dihuang decoction,” “pathogenesis of depression,” and “pharmacological action and mechanism of depression.

**Results:**

This paper reviewed the traditional applications and dosages of the *R. glutinosa-Lilium* as documented in Chinese medical classics, thereby establishing a foundation for the contemporary development and clinical application of the classical formula Baihe Dihuang Decoction. Additionally, recent years have seen a comprehensive review of the pharmacological effects and mechanisms of *R. glutinosa-Lilium* and its principal metabolites in the context of depression.

**Conclusion:**

This paper has reviewed the active metabolites of *R. glutinosa-Lilium* and demonstrated its efficacy in the treatment of depression, as well as its role in modulating the underlying mechanisms of the disorder. The findings aim to serve as a reference for further research into the mechanisms of depression, its clinical applications, and the development of novel therapeutic agents.

## 1 Introduction

Depression, being a prevalent mental disorder, is characterized by a range of clinical manifestations including but not limited to low mood, sluggish cognitive processing, sleep disturbances, social withdrawal, reduced motivation, and in severe cases, self-injurious behavior (Filatova et al., 2021). The COVID-19 pandemic resulted in a global increase of 28% in depression cases in 2020, with young people in particular suffering (Sljivo and Kulenovic, 2023; Blomqvist et al., 2023). At present, the etiology of depression remains incompletely comprehended, albeit the most notable hypotheses include an imbalance of monoamine neurotransmitter imbalance (Hirschfeld, 2000), decreased concentration of neurotrophins (Duman and Monteggia, 2006), inflammation and oxidative stress (Yang et al., 2020), disorder of Hypothalamus-Pituitary-Adrenal (HPA) axis (Yu et al., 2023), intestinal flora imbalance (Lach et al., 2018), mitochondrial dysfunction (Buttiker et al., 2022), etc. Despite the efficacy of selective serotonin reuptake inhibitors, tricyclic antidepressants, and other Western pharmacological interventions in treating depression, they are associated with certain drawbacks, including suboptimal therapeutic outcomes, prolonged duration of action, significant adverse effects, and high expenses (Kovich et al., 2023; Stachowicz and Sowa-Kucma, 2022). As a result, it is imperative to find new depression-treating drugs.

Traditional Chinese medicine (TCM) compound prescriptions offer numerous advantages in the treatment of depression, such as the incorporation of multiple metabolites, pathways, and targets, with a notable emphasis on drug compatibility (Xiong et al., 2022; Liu et al., 2023; Yang et al., 2022). Compared with the compound of TCM, drug pairing is a relatively fixed form of two-flavor drug in clinical use, which is more conducive to clarifying the interaction mechanism between drugs and the mechanism of action of drugs on the body (Tao et al., 2018; Song et al., 2017; Wang B. H. et al., 2021). *Rehmannia glutinosa-Lilium* is a classic antidepressant pair with pharmacological effects such as antidepressant, anxiolytic, anti-inflammatory, etc (Ma et al., 2019; Zhao et al., 2022; Chi et al., 2019). *Rehmannia glutinosa DC.*, a member of the Scrophulariaceae family, is a traditional Chinese medicinal botanical drug that possesses the ability to alleviate heat, promote blood cooling, and enhance yin and fluid nourishment (Geng, 2022). Contemporary pharmacological studies have demonstrated its antioxidant, anti-inflammatory, bacteriostatic, antidepressant, sedative, and hypnotic properties (Li et al., 2022; Yan et al., 2021; Liu C et al., 2017). *Lilium lancifolium Thunb.* is a dry, fleshy scale leaf of the Liliaceae family, which is a Chinese medicinal botanical drug that nourishes the lungs and clears the mind and calms the mind (He D et al., 2022). Modern pharmacology has found that it has antioxidant, anti-inflammatory, bacteriostatic, antidepressant, sedative, and hypnotic effects (Zhou et al., 2021; Pan et al., 2017; Sim et al., 2020). At present, there are many studies on *R. glutinosa* and *Lilium* single medicine, and clinical studies show that Baihe Dihuang Decoction as a Chinese medicine’s compound prescriptions have a good effect on depression treatment, but its main active metabolites and pharmacological mechanism have not been described. This article reviews the antidepressant active metabolites and their mechanism of action in the combination of *R. glutinosa-Lilium*, for the purpose of providing references for research on depression’s mechanism of action, clinical application, and new drug development.

## 2 Methods of data acquisition

To ensure a comprehensive and systematic review of the existing literature on Rehmannia glutinosa-Lilium, a meticulous search strategy was implemented. A comprehensive literature search was conducted as of 29 September 2024, utilizing databases such as PubMed, CNKI, Wanfang Data, Baidu Scholar, and Google Scholar. Additionally, classical texts on Chinese herbal medicine, the Chinese Pharmacopoeia, as well as doctoral and master’s theses, were included in the collected materials.

The search terms were carefully selected to encompass the broad spectrum of research areas relevant to Rehmannia glutinosa-Lilium. The search employed specific terms including “*R. glutinosa*,” “*Lilium*,” “Baihe Dihuang decoction,” “Traditional uses of *R. glutinosa-Lilium*,” “Chemical composition of *R. glutinosa*,” “chemical composition of Lilium” “neurotransmitters and depression,” “Brain-derived neurotrophic factor and depression,” “oxidative stress and depression,” “glutamic acid and depression,” “the hypothalamus-pituitary-adrenal and depression,” “intestinal microorganisms and depression,” “application of Baihe Dihuang decoction,” “pathogenesis of depression,” and “pharmacological action and mechanism of depression.

The inclusion criteria for the studies were as follows: 1) studies that report traditional uses of *R. glutinosa-Lilium*, 2) studies that report active metabolites in antidepressants of Rehmannia glutinosa-Lilium, 3) research the effect of the active metabolites in *R. glutinosa-Lilium* on depression. Studies not directly pertinent to these areas and *in vitro* experimental studies are excluded, and records are subsequently screened based on title and abstract to identify those meeting the inclusion criteria. The full articles are then obtained for further relevance assessment. Data extraction concentrates on the historical application of *R. glutinosa-Lilium*, its chemical composition, and its role in the treatment of depression. Finally, the extracted data are synthesized and prepared for comprehensive analysis in the review.

## 3 Traditional uses of *Rehmannia glutinosa-Lilium*

The synergistic effects of *R. glutinosa* and *Lilium* have been found to be efficacious in the treatment of a diverse range of ailments such as depression, climacteric syndrome, anxiety, insomnia, cancer, hypertension, and others (Qing et al., 2023; Zhu and Xie, 2022). The earliest recorded herbal literature of *R. glutinosa* and *Lilium* is *Shen Nong Ben Cao Jing* of the Eastern Han Dynasty (Wu, 1963). Nevertheless, the composition of the Baihe Dihuang Decoction, which includes both *R. glutinosa* and *Lilium*, was first recorded in *Jin Kui Yao Lun* authored by Zhang Zhongjing (He and He, 2005). The treatment and preparation methods of Baihe Dihuang Decoction, as documented by physicians in previous dynasties, have remained largely consistent, with the exception of variations in the quantities of *Lilium* and raw *R. glutinosa* juice utilized. During the Tang Dynasty, Sun Simiao, a physician, modified the dosage of *R. glutinosa* juice to 2 L in *Bei Ji Qian Jin Yao Fang*, a treatment for Lily disease and irregular menstruation (Gao and Shen, 2008). Lily disease is clinically manifested as anxiety and depression, and depression is the main clinical manifestation of Lily disease (Junjie et al., 2024). Similarly, in the Song Dynasty, Pang Anshi altered the dosage of *Lilium* to ten and the dosage of *R. glutinosa* juice to half a liter in the treatment of Lily disease, as documented in *Shang Han Zong Bing Lun* (Zou and Liu, 1989). The documentation pertaining to the primary administration, formulation, and application of Baihe Dihuang Decoction in *Jin Kui Fang Lun Yan Yi* during the Yuan Dynasty aligns with the principles outlined in Zhang ZhongJing’s theory (Zhou and Wang, 1993). In *Ben Cao Hui Yan*, Ni Zhumu, a physician during the Ming Dynasty, introduced a modification to the administration of *R. glutinosa* by increasing the dosage to eight taels (Zheng, 2005). The effectiveness and preparation techniques documented in other medical texts from the Ming Dynasty, such as *Yi Zong Bi Du* (Gu, 2005) and *Zu Ji* (Da, 1987) as well as those from the Qing Dynasty, including *Jin Kui Fang Ge Kuo* (Chen and Chen, 1963), *Zhang Shi Yi Tong* (Sun and Wang, 2005), *Wen Re Jing Wei* (Lu, 1997) have not changed much compared with the *Jin Kui Yao Lun*. Presently, Baihe Dihuang Decoction has been included in the initial group of ancient traditional formulas and is predominantly employed in the management of depression (Ma et al., 2019) (Table 1).

**TABLE 1:** Traditional uses of *Rehmannia glutinosa-Lilium*.

Dynasty of ancient China	Classic medica books	Traditional application	Dose change	References
The Eastern Han Dynasty	*Jin Kui Yao Lun*	Lily disease	7 lilies, 1 L of raw *Rehmannia glutinosa*	He and He (2005)
The Tang Dynasty	*Bei Ji Qian Jin Yao Fang*	Lily disease, Irregular menstruation	7 lilies, 2 L of raw *Rehmannia glutinosa*	Gao and Shen (2008)
The Song Dynasty	*Shang Han Zong Bing Lun*	Lily disease	10 lilies, half a liter of raw *Rehmannia glutinosa*	Zou and Liu (1989)
The Yuan Dynasty	*Jin Kui Fang Lun Yan Yi*	Lily disease	7 lilies, 1 L of raw *Rehmannia glutinosa*	Zhou and Wang (1993)
The Ming Dynasty	*Ben Cao Hui Yan*	Lily disease	7 lilies, 1 L of raw *Rehmannia glutinosa*	Zheng (2005)
The Ming Dynasty	*Yi Zong Bi Du*	Lily disease	7 lilies, eight taels of raw *Rehmannia glutinosa*	Gu (2005)
The Ming Dynasty	*Zu Ji*	Lily disease	7 lilies, 1 L of raw *Rehmannia glutinosa*	Da (1987)
The Qing Dynasty	*Jin Kui Fang Ge Kuo*	Lily disease	7 lilies, 1 L of raw *Rehmannia glutinosa*	Chen and Chen (1963)
The Qing Dynasty	*Zhang Shi Yi Tong*	Lily disease	7 lilies, 1 L of raw *Rehmannia glutinosa*	Sun and Wang (2005)
The Qing Dynasty	*Wen Re Jing Wei*	Lily disease	7 lilies, 1 L of raw Rehmannia glutinosa	Lu (1997)

## 4 *Rehmannia glutinosa-Lilium* active metabolites in antidepressants

The efficacy of TCM in treating depression has garnered the attention of scholars worldwide, prompting them to explore TCM’s compound prescription. Recently, many Chinese proprietary medicines with good antidepressant properties have been discovered, including Chaihu Shugan Powder (Fan et al., 2023), Yueju Pill (Ren and Chen, 2017), Baihe Dihuang Decoction (Xue X. Y. et al., 2022), Kaixin Powder (Xu F. et al., 2023) and Sini Powder (He X et al., 2022), etc. With further in-depth study, it is found that the material basis of antidepressant effect in TCM compound prescription is the active metabolites of TCM (Zhang H. et al., 2021; Deng et al., 2022; Yang et al., 2023). *Rehmannia glutinosa and Lilium* are both medicinal and edible plants, as a commonly used antidepressant pair, has a good improvement effect on depression (Table 2). Baihe Dihuang Decoction was identified by liquid mass spectrometry (LP-MS) with 94 chemical metabolites, including 33 metabolites into blood and 9 metabolites into brain (Wu et al., 2021). The liquid chromatography-mass spectrometry (LC-MS) technique was employed to analyze the decoction of *Lilium*, *Rehmannia* and Baihe Dihuang Decoction, which revealed the presence of 36 novel compounds in the Baihe Dihuang Decoction that were not detected in the individual decoctions of *Lilium* and *Rehmannia*, and the antidepressant active metabolites verbascoside only existed in the co-decoction (Mao et al., 2024). The identified metabolites were correlated with depression, and it was determined that saponins, phenylpropanoids, iridoid terpenoids, flavonoids, alkaloids, and phenylethanol glycosidesmay constitute the primary active metabolites in the therapeutic management of depression (Table 3).

**TABLE 2:** Ameliorating effect of Baihe Dihuang Decoction on depression.

Extracts/metabolites	Controls	Model	Animal/cell	Dose range tested	Duration	Key indicators	References
Baihe Dihuang Decoction	Fluoxetine hydrochloride (18 mg/kg)	Solitary feeding and chronic unpredictable mild stress stimulation (CUMS)	Male SD rat	3.75, 7, 15 g/kg	28 days	Firmicutes↑,Bacteroidota↓, V (Zhao et al., 2021a) oteobacteria↓, Cyanobacteria↓	Feng et al. (2024)
Baihe Dihuang Decoction	Rolipram (0.1 mg/mL)	Chronic unpredictable stress (CUS)	Male ICR mice	0.3, 0.6, 1.2 g/mL	35 days	ACTH↓CORT↓, cAMP↑	Zhou et al. (2023)
Baihe Dihuang Decoction	venlafaxine (13.5 mg/kg)	Chronic restraint stress combined with subcutaneous injection of corticosterone	SD rats	4, 16 g/kg	21 days	IL-1β↓, IL-6↓, IL-18↓, NLRP3↑, ASC↑, Caspase-1↑	Zhao et al. (2021b)
Baihe Dihuang Decoction	Fluoxetine hydrochloride (20 mg/kg)	CUMS	Male SD rat	90 g/kg	28 days	IL-1β↓, IL-6↓, TNF-α↓, Glu↓, IL-10↑, 5-HT↑, DA↑, NE↑, GABA↑	Pan et al. (2023)

**TABLE 3:** The main antidepressant metabolites of *Rehmannia glutinosa-Lilium.*

Classify	Metabolites	Structural formula	Chemical formula	CAS	Source
Saponins	Regaloside A	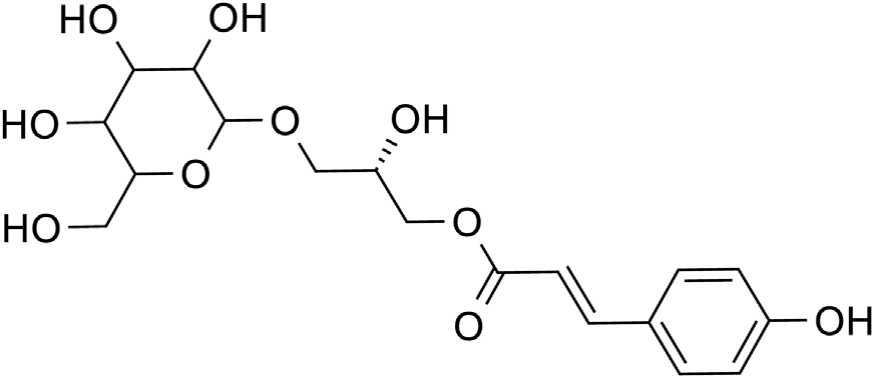	C_18_H_24_O_10_	114,420-66-5	*Lilium*
	Regaloside B	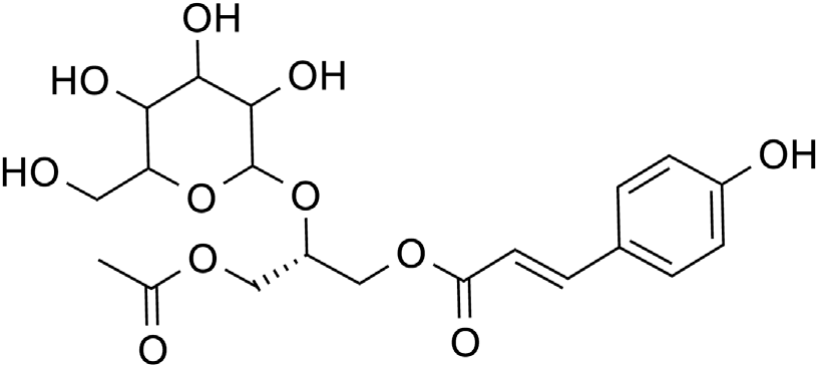	C_18_H_24_O_11_	114,420-67-6	*Lilium*
	Regaloside C	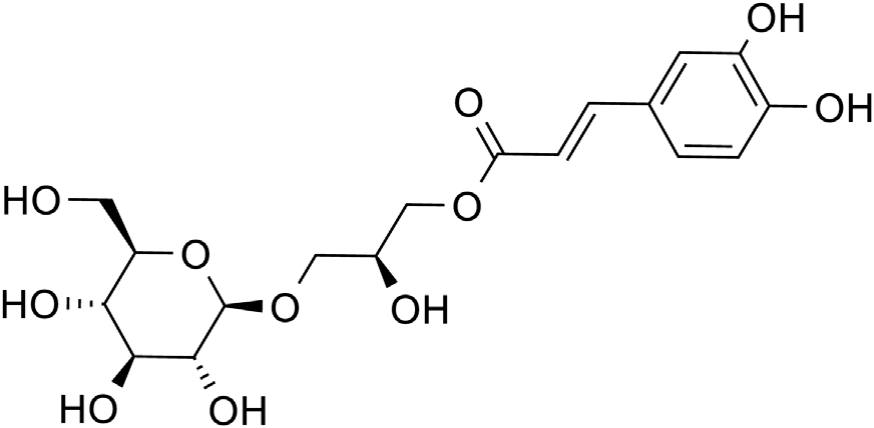	C_18_H_24_O_11_	117,591-85-2	*Lilium*
Phenylpropanoids	Chlorogenic acid	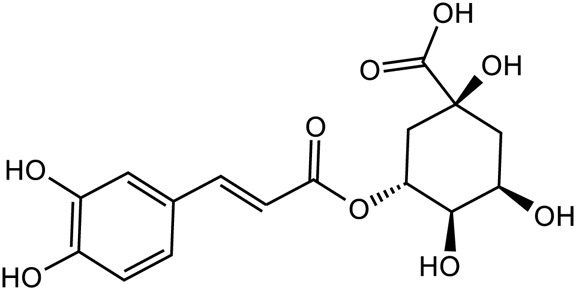	C_16_H_28_O_9_	1,049,703-62-9	*Lilium*
	Ferulic acid	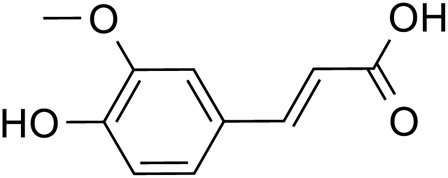	C_10_H_10_O_4_	1,135-24-6	*Lilium*
Iridoid terpenoids	Aucubin	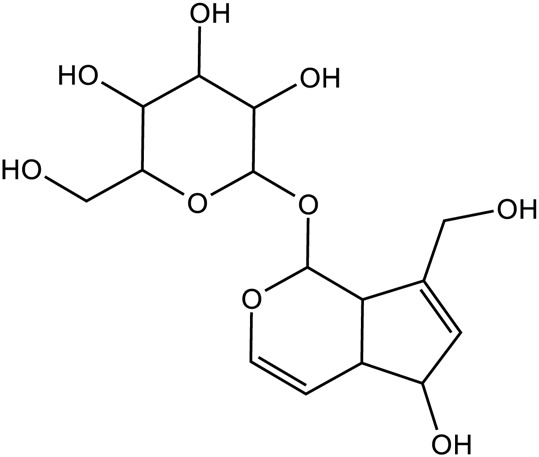	C_15_H_22_O_9_	479-98-1	*Rehmannia glutinosa*
	Ajugol	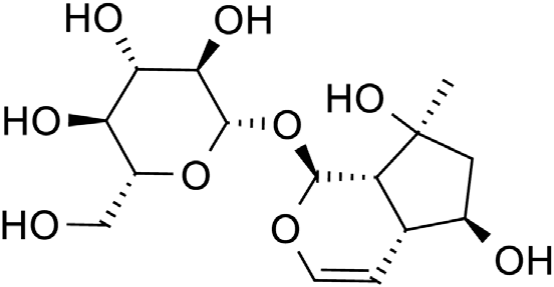	C_15_H_24_O_9_	52,949-83-4	*Rehmannia glutinosa*
	Acteoside	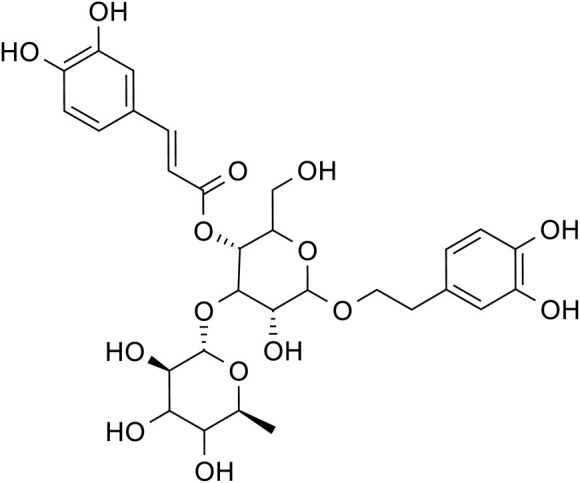	C_29_H_36_O_15_	61,276-17-3	*Rehmannia glutinosa*
	Catalpol	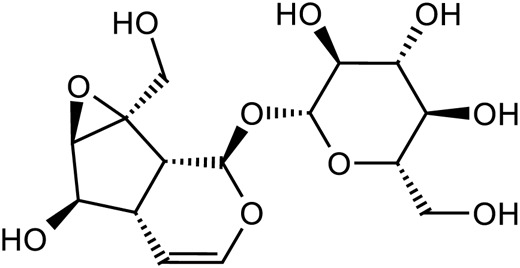	C_15_H_22_O_10_	2,415-24-9	*Rehmannia glutinosa*
	Dihydrocatalpol	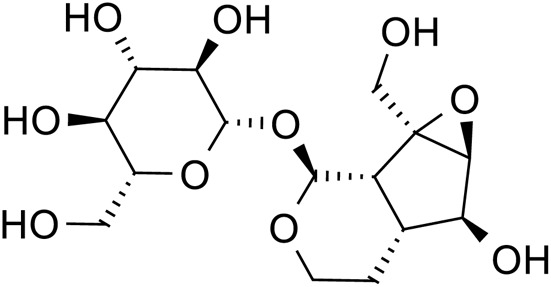	C_15_H_24_O_10_	6,736-86-3	*Rehmannia glutinosa*
	Geniposide	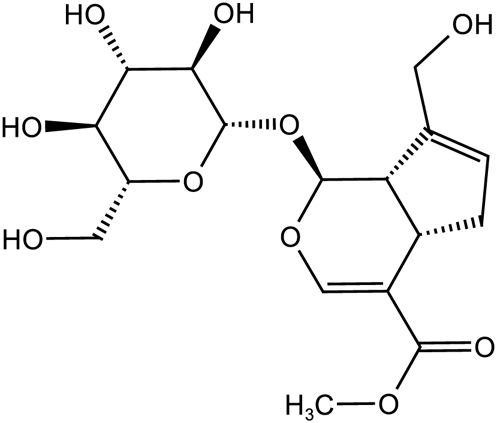	C_17_H_24_O_10_	24,512-63-8	*Rehmannia glutinosa*
Flavonoids	Quercetin	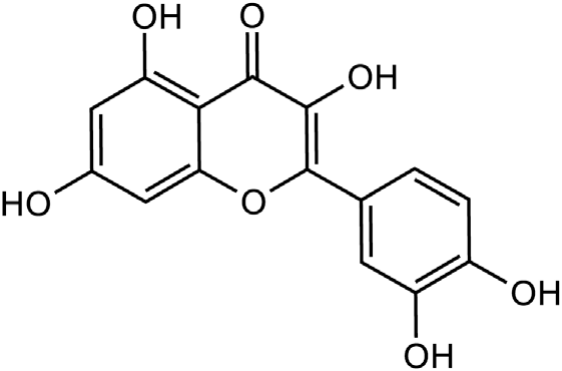	C_15_H_10_O_7_	117-39-5	*Lilium*
	Luteolin	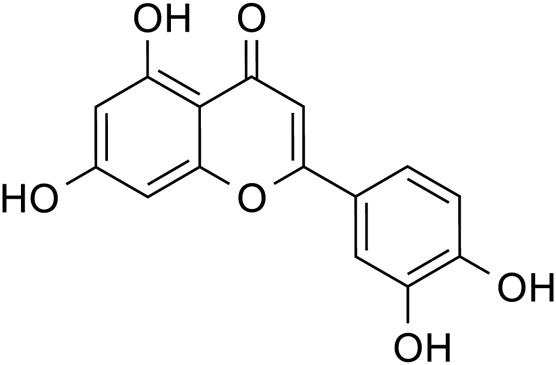	C_15_H_10_O_6_	491-70-3	*Lilium*
	Kaempferol	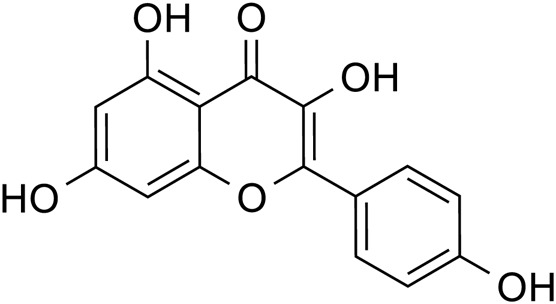	C_15_H_10_O_6_	520-18-3	*Lilium*
	Apigenin	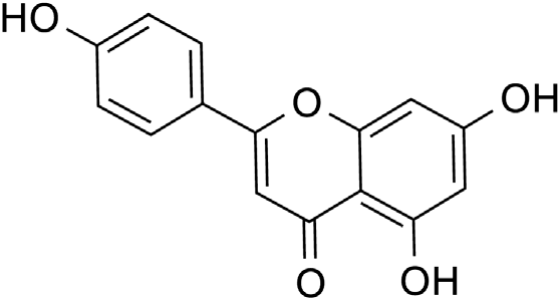	C_15_H_10_O_5_	520-36-5	*Lilium*
Alkaloid	Colchicine	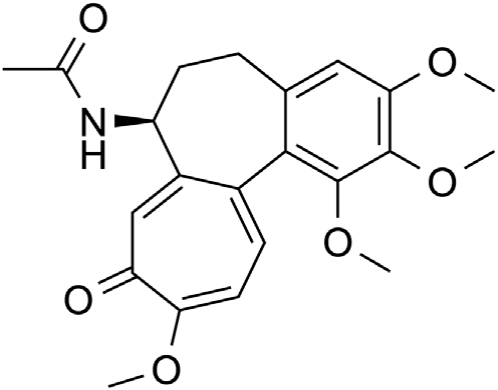	C_22_H_25_NO_6_	64-86-8	*Lilium*
	Berberine	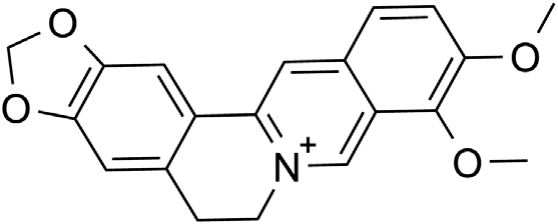	C_17_H_17_N	2086-83-1	*Lilium*
Phenylethanol Glycosides	Rhmannioside D	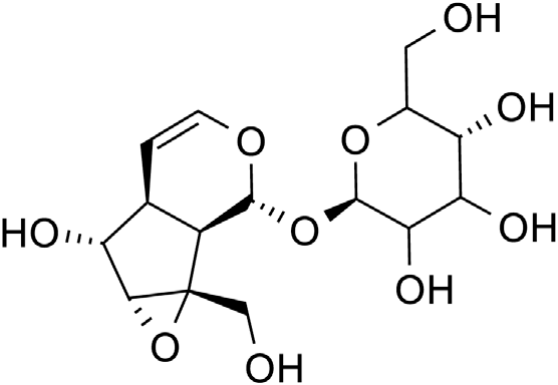	C_27_H_42_O_20_	81,720-08-3	*Rehmannia glutinosa*
	Hyperoside	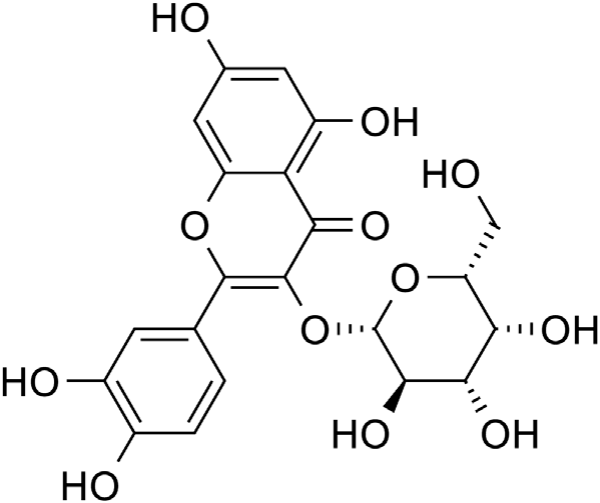	C_21_H_20_O_12_	482-36-0	*Rehmannia glutinosa*

## 5 Antidepressant mechanism of *Rehmannia glutinosa-Lilium*

### 5.1 Effects of active metabolites of *Rehmannia glutinosa-Lilium* on the function of monoamine neurotransmitters

Neurotransmitters play a crucial role in facilitating signal transmission between neurons and effectors within the body (Hu and Wu, 2023). Monoamine neurotransmitters are central neurotransmitters, including catecholamine and indoleamine, the former is a neurotransmitter based on catecholamine, including norepinephrine (NE), epinephrine and dopamine (DA), while the latter is composed of indole and ethylamine, which mainly refers to 5-hydroxytryptamine (5-HT) (Wu, 2023). The occurrence of depression is associated with the modulation of monoamine neurotransmitter levels, and when a decrease in the levels of 5-HT, NE, and DA may have an impact on the emotional state of individuals (El et al., 2010; Perez-Caballero et al., 2019).

**FIGURE 1:**
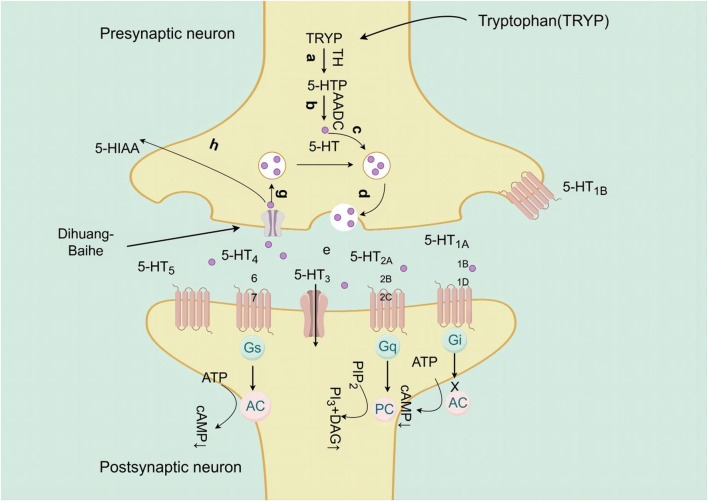
The active substances in *Rehmannia glutinosa-Lilium* inhibits 5-HT reuptake in nerve endings and cell bodies.

Catalpol, the chemical metabolites, found in *R. glutinosa*, possesses a significant concentration of iridoids and displays a multitude of pharmacological properties, including but not limited to antidepressant, cognitive-enhancing, and neuroprotective effects (Zhang and Liu, 2019). Treatment with catalpol (5, 10, or 20 mg/kg) for 14 days reduced mice’s depressive-like behavior in a depression model, and it was found that catalpol increased the content of 5-HT and 5-hydroxyindoleacetic acid (5-HIAA) in mice’s brains, while exhibiting minimal influence on the levels of NE and DA. This study to indicate that catalpol has an antidepressant-like effect and that its action may be mediated by the central serotonergic system (Wang et al., 2014). The phenylethanol glycoside acteoside, extracted from *Radix Rehmanniae*, exhibits many pharmacological properties, such as antidepressant properties, antitumor properties, anti-inflammatory properties, neuroprotective properties, etc (Ge et al., 2023). Current studies have have demonstrated that acteoside substantially elevates the serum concentrations of 5-HT, GABA, and DA in depressed mice, and the mechanism underlying the antidepressant effects of acteoside is believed to involve the augmentation of monoamine neurotransmitters, the attenuation of pro-inflammatory cytokines, and the restoration of neurotransmitter levels (Xue X. et al., 2022). Recent studies have revealed that the primary metabolites of *Lilium* are saponins, which exhibit antidepressant, antioxidant, anti-inflammatory, antibacterial, and regulatory properties on the cerebral and gut axis (Sun et al., 2022). Administration of intragastric *Lilium* saponins to mice with depression resulted in a reduction of depressive-like behavior and a decrease in body temperature (Wang, 2014). Moreover, the administration of *Lilium* extracts resulted in elevated levels of DA and 5-HT, thereby restoring the function of monoamine neurotransmitters in rats with depression (Guo et al., 2009). Gallic acid, a ubiquitous phenolic acid in nature, is a crucial bioactive metabolites of *Lilium*, exhibiting anti-aging, antioxidant, anti-inflammatory, and other therapeutic properties (Zhang and Ma, 2020; Wang P. et al., 2023). Administration of 60 mg/kg gallic acid reduced depressive behavior in depressive model mice, and its antidepressant action is mediated by increasing not only 5-HT levels in the synaptic space, but also catecholamine levels in the brain (Can et al., 2017). Berberine, the principal bioactive metabolites of *Lilium* alkaloids, exhibits a range of pharmacological effects including antibacterial, anti-inflammatory, antiviral, lipid-modulating, hypoglycemic, antiarrhythmic, antihypertensive, immunomodulatory, and antitumor properties (Chen et al., 2023; Zhao L. et al., 2023; Tan et al., 2023). Berberine has been observed to significantly decrease the resting time of TST and FST in mice with depression, while concurrently elevating the levels of NE and 5-HT in the hippocampus and prefrontal cortex, and the mechanism of action of berberine is believed to be linked to the regulation of monoamine neurotransmitters in the brain (Peng et al., 2007) (Figure 1; Table 4).5-HT may contribute to the pathophysiology of depression via the cAMP/PKA/CREB signaling pathway, which is mediated by the 5-HT1A receptor (Brites and Fernandes, 2015). The elevated concentration of the psamine transporter (DAT) enhances the reuptake rate of DA at synaptic terminals, resulting in a reduction of DA levels in the synaptic cleft and subsequently contributing to depressive symptoms (Zaaijer et al., 2015). 5-HT and NE interact with G protein-coupled receptors (GPCRs) to facilitate neural transmission and generate electrical signals that modulate emotional responses (Yang et al., 2018). Consequently, *R. glutinosa-Lilium* may modulate depressive symptoms by enhancing the secretion and synthesis of neurotransmitters such as 5-HT, DA, and NE.

**TABLE 4:** Active substances regulating monoamine neurotransmitters in *Rehmannia glutinosa-Lilium.*

Extracts/metabolites	Controls	Model	Animal/cell	Dose range tested	Duration	Key indicators	References
Catalpol	Fluoxetine hydrochloride (10 mg/mL)	Reserpine	Male Kunming mice	5, 10, 20 mg/kg	14 days	5-HT↑, 5-HIAA↑	Wang et al. (2014)
Acteoside	Fluoxetine (20 mg/mL)	CUMS	Male C57BL/6 mice	60 mg/kg	21 days	5-HT↑, GABA↑, DA↑	Xue X. Y. et al. (2022)
*Lilium* saponins	Fluoxetine (0.04 g/kg)	Reserpine	Female, male Kunming mice	25, 50, 100 mg/kg	7 days	Reduce the change of body temperature in mice	Wang (2014)
	Fluoxetine (2 mg/mL)	CUMS	Male SD rat	12, 24, 48 mg/kg	21 days	DA↑, 5-HT↑	Guo et al. (2009)
Gallic acid	Fluoxetine (30 mg/mL)	Depression	Male BALB/c mice	30, 60 mg/kg	4 days	5-HT↑, DA↑	Can et al. (2017)
Berberine	Fluoxetine (20 mg/mL)	Depression	Male ICR albino mice	10, 20 mg/kg	30 min	NE↑, 5-HT↑	Peng et al. (2007)

**Note:** 5-HT, 5-hydroxytryptamine; CUMS, chronic unpredictable mild stimulation; 5-HIAA, 5-hydroxyindole acetic acid; GABA, γ-aminobutyric acid; DA, Dopamine.

### 5.2 Brain-derived neurotrophic factor levels in response to *Rehmannia glutinosa-Lilium* active metabolites

Brain-derived neurotrophic factor (BDNF) is a crucial neurotrophic factor in the brain that plays a pivotal role in the growth, survival, and synapse formation of neurons that are linked to emotional and cognitive functions (Lima et al., 2019). According to a study of depression patients, the level of BDNF decreased with increasing severity of the disease, and the more serious the condition, the lower the level of BDNF (Du et al., 2023; Lee et al., 2007). There are two receptors for BDNF, one high-affinity receptor that binds tyrosine kinase receptor B and another low-affinity receptor that binds neurotrophic factor (p75 NTR). BDNF participates in the pathophysiological process of depression mainly through the induction of intracellular tyrosine residue autophosphorylation and receptor dimerization by binding to TrkB (Liu and Wang, 2015). The primary routes through which phosphorylated TrkB initiates downstream signaling cascades predominantly encompass the PI3K/AKT pathway, MAPK pathway, and PLCγ/PKC pathway. These pathways facilitate enhanced synaptic plasticity, improved neuronal growth and survival, and ultimately provide neuroprotection and nutritional support to the nerves (Wang et al., 2020; Mosiolek et al., 2021).

**FIGURE 2:**
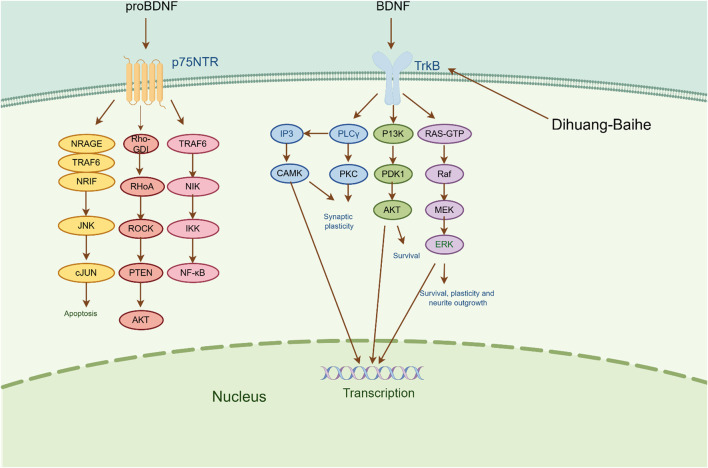
The active metabolites of *Rehmannia glutinosa-Lilium* can regulate brain-derived neurotrophic factor. (BDNF, Brain-derived neurotrophic factor; TrkB, Tyrosine kinase receptor B; P75NRT, Neurotrophin P75 receptor; PLCγ, Phospholipase Cγ; PI3K, Phosphatidylinositol 3 kinase; Raf, Raf kinas).

According to the experiment, catalpol significantly increased PI3K, Akt, Nrf2, HO-1, TrkB, BNDF, and other gene and protein expression in rats modeled by CUMS, and confirmed that PI3K/Akt/Nrf2/HO-1 signaling pathways were upregulated by catalpol’s antidepressant mechanism on depression, improving hippocampal neuroprotection and antioxidant levels (Wang J et al., 2021). Phenolic acids are one of the main active metabolites of lilies, in which Regaloside A in *Lilium* saponins plays a role in various antidepressant compound prescriptions (Luo et al., 2017). Following treatment with Regaloside A, there was an increase in the cell survival rate and phosphorylation levels of BDNF, TrkB, PI3K, and Akt, which is postulated that Regaloside A exerts antidepressant effects via the BDNF-TrkB pathway (Yuan et al., 2021). Contemporary pharmacological research has determined that Rehmanin D possesses the capability to mitigate PC-12 cell impairment caused by elevated levels of cortisol, and the effect is attributed to its potential to augment BDNF expression and elicit anti-apoptotic responses via the BDNF-TrkB pathway, ultimately safeguarding nerve cells and manifesting antidepressant properties (Zhang et al., 2022). After chlorogenic acid treatment, the nerve damage score and brain water content of mice decreased, BDNF, NGF, 5-HT, and 5-HIAA proteins were upregulated, while pro-inflammatory cytokines iNOS, IL-6, TNF-α, NLRP3, and IL-1β were significantly downregulated (Liu et al., 2021) (Figure 2; Table 5). BDNF specifically binds to the TrkB receptor, thereby activating downstream signaling pathways such as PI3K/Akt, MAPK, and cAMP, among others (Fries et al., 2023). This interaction enhances the release of presynaptic neurotransmitters, facilitating nerve signal transmission (Li et al., 2024). Consequently, *R. glutinosa-Lilium* has been shown to elevate BDNF levels, thereby supporting normal neuronal function and promoting emotional recovery as well as the improvement of cognitive function.

**TABLE 5:** An active metabolites in *Rehmannia glutinosa-Lilium* that regulates the level of a brain-derived neurotrophic factor.

Extracts/metabolites	Controls	Model	Animal/cell	Dose range tested	Duration	Key indicators	References
Catalpol	Fluoxetine hydrochloride (10 mg/mL)	CUMS	Male SD rats	10 mg/kg	35 days	PI3K↑, Akt↑, Nrf2↑, HO-1↑, TrkB↑, BDNF↑	Wang H. H. et al. (2021)
Regaloside A	Mock	CORT	SH-SY5Y cells	5, 15, 25, 50, 75, 100 μmol/L	24 h	Akt↑, BDNF↑, TrkB↑, PI3K↑	Yuan et al. (2021)
Rehmannia Glycoside D	Fluoxetine (0.3 μmol/L)	CORT	PC-12 cells	5, 10, 20 μmol/L	24 h	BDNF↑, TrkB↑	Zhang et al. (2022)
Chlorogenic acid	0.5% sodium carboxymethyl cellulose (20, 50, 100 mg/kg)	Aβ	Male Kunming mice	20, 50, 100 mg/kg	21 days	BDNF↑, NGF↑, 5-HT↑, 5-HIAA↑, iNOS↓, IL-6↓, TNF-α↓, NLRP3↓, IL-1β↓	Liu et al. (2021)

**Note:** PI3K, Phosphatidylinositol-3-kinase; CUMS, chronic unpredictable mild stimulation; Nrf2, Nuclear factor E2-related factor 2; HO-1, Heme oxygenase 1; CORT, cortisol; TrkB, Tyrosine kinase receptor B; BDNF, Brain-derived neurotrophic factor; Akt, Protein kinase B.

### 5.3 *Rehmannia glutinosa-Lilium* metabolites’ effects on inflammation and oxidative stress

Oxidative stress refers to the state of cellular imbalance resulting from the overproduction of reactive oxygen species (ROS) and the insufficient antioxidant capacity of cells (Sies, 2015). This condition can lead to various pathological processes, including but not limited to inflammation, neurodegeneration, tissue damage, and cell death, when ROS production exceeds the antioxidant response (Bhatt et al., 2020; Redza-Dutordoir and Averill-Bates, 2016; Xing et al., 2024a). The pathophysiology of depression is strongly influenced by oxidative stress and inflammation, the body is stimulated by stress, the redox balance is broken, and the body’s antioxidant enzyme function changes abnormally, producing excess ROS, pro-inflammatory factors are released, and the inflammatory response is activated, ultimately leading to disturbances in the structure and function of biological macromolecules and proteins in nerve cells, culminating in the manifestation of depression (Kohler et al., 2016; Pandey et al., 2018; Vavakova et al., 2015; Zhang, 2018). Clinical studies have provided empirical evidence indicating that individuals suffering from inflammatory diseases are more likely to experience depression (Beurel et al., 2020). Furthermore, an elevation in the levels of pro-inflammatory cytokines, specifically IL-1β, IL-6, and TNF-α, is positively correlated with the severity of depressive symptoms (Neupane et al., 2022; Boucas et al., 2022). Moreover, inhibiting ROS and malondialdehyde (MDA) and increasing antioxidant enzymes like superoxide dismutase and catalase (CAT) can alleviate depression symptoms (Lindqvist et al., 2017).

**FIGURE 3:**
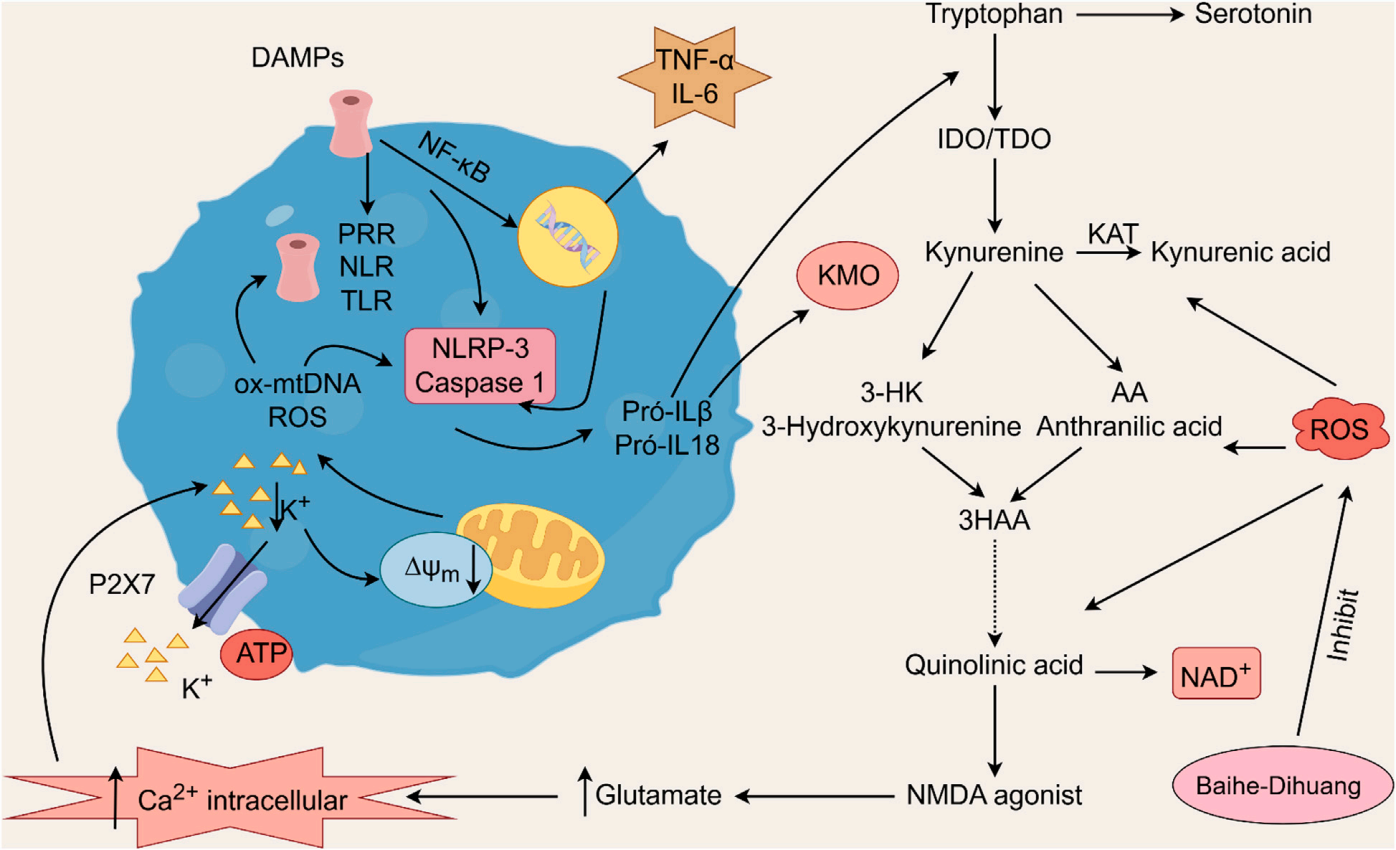
The activation of pattern recognition receptors (PRRs) triggers the NLRP3 inflammasome and caspase-1, leading to interleukin-1 beta (IL-1β) and interleukin-18 (IL-18) activation. Oxidized mitochondrial DNA (ox-mtDNA) and mitochondrial reactive oxygen species (ROS) also activate the inflammasome. Additionally, nuclear factor kappa-light-chain-enhancer of activated B cells (NF-κB) promotes the production of tumor necrosis factor alpha (TNF-α) and interleukin-6 (IL-6). The proinflammatory cytokines IL-1β and IL-18 further activate the enzymes indoleamine 2,3-dioxygenase (IDO) and tryptophan 2,3-dioxygenase (TDO) within the kynurenine pathway, facilitating the degradation of tryptophan into kynurenine. These two cytokines further activate kynurenine 3-monooxygenase (KMO), the enzyme responsible for directing the degradation of kynurenine into 3-hydroxykynurenine (3HK) and quinolinic acid, both of which are neurotoxic agents, rather than into kynurenic acid, a neuroprotective agent. Kynurenic acid functions as an NMDA receptor agonist and enhances glutamate levels, subsequently increasing intracellular calcium concentrations. This process results in the excessive production of reactive oxygen species (ROS) via the kynurenine pathway.

Geniposide, an iridoid discovered in *R. glutinosa*, also has demonstrated antidiabetic, antioxidant, antidepressant, and neuroprotective properties (He et al., 2023; Kimura et al., 2023; Li et al., 2020). The current investigation provides evidence that the regulation of GLP-1R/AKT by geniposide effectively mitigates depressive behavior induced by repeated inhibitory stress (RRS) and hippocampal neuronal apoptosis in mice, concomitantly decreasing the content of pro-inflammatory cytokines IL-1β and TNF-α (Zhao et al., 2018). The ethanol extract derived from *Lilium* exhibits a specific inhibitory effect on the nuclear factor κ B (NF- κ B) signal pathway, which is induced by inhibitor kappa B kinase β (IKK β), thereby exerting an anti-inflammatory effect and the main bioactive metabolites in the *Lilium* alcohol extract were identified as quercetin, luteolin, and kaempferol through high performance liquid chromatography (HPLC) (Han et al., 2018). This observation indicates that lilies may mitigate oxidative stress by modulating glutamate metabolism, which subsequently activates the Nrf-2 signaling pathway (Xing et al., 2024b). Quercetin, luteolin, and kaempferol are flavonoids in Lilium, which have potent antioxidant activity (Xu et al., 2022). The administration of Quercetin has been observed to yield a significant reduction in anxiety and depression in mice that have been subjected to chronic unpredicted stress (CUS)-induced depression and quercetin has been observed to decrease the expression of oxidative stress markers and pro-inflammatory cytokines in hippocampal neurons, thereby conferring protection to the mouse brain against oxidative and inflammatory stress (Mehta et al., 2017). The administration of luteolin to CUMS mice leads to a significant increase in the activation of SOD and GSH-Px in brain tissue, a reduction in MDA levels, and inhibition of neuronal oxidative stress (Liu et al., 2013). Kaempferol activates the AKT/catenin cascade in the prefrontal cortex of CSDS mice, thereby augmenting its antioxidant and anti-inflammatory properties (Gao et al., 2019). Notably, a neuroinflammatory response is triggered by the activation of microglia and subsequent release of pro-inflammatory cytokines (Zhang et al., 2016). Under normal circumstances, microglia (M0) in the central nervous system are in a quiescent state and play the role of “immune surveillance,” while microglia in the pathological state come to life and release a series of cytokines, which participate in the occurrence and development of neuroinflammation (Xu et al., 2020). The continuous activation of classical activated microglia (M1) will produce excessive inflammatory factors and oxidative stress, causing damage to nerve cells and leading to aggravation of the disease, while alternative activated microglia (M2) can promote tissue repair and regeneration and play a neuroprotective role (Kwon and Koh, 2020; Wolf et al., 2017; Chen et al., 2024). Thus, the inhibition of M1 microglia proliferation can lead to an improvement in depressive symptoms and a prevention of neuroinflammation. Compared with the model group, the Rehmannia glycoside D-group exhibited a reduction in the levels of pro-inflammatory cytokines IL-6 and IL-1β released by M1 microglia, and an increase in the levels of anti-inflammatory cytokines IL-4 and IL-10 released by M2 microglia, which may be attributed to the inhibition of microglial transformation from M2 to M1 (Wang H. H. et al., 2021). As measured by CUMS, Catalpol not only increased hippocampal SOD, CAT, GSH-Px, GST, GST and GSH levels in rats, but also inhibit microglial polarization of the M1 phenotype and reduce the expression of IL-1β, TNF-α and iNOS (Wang Y. T. et al., 2021) (Figure 3; Table 6). Oxidative stress can enhance the activity of the rate-limiting enzyme in tryptophan metabolism by promoting neuroinflammation, leading to increased production of quinolinic acid and stimulating microglia to express kynurenine-3-monooxygenase (KMO, resulting in the conversion of kynurenine to the neurotoxic metabolite quinolinic acid (Vaglio-Garro et al., 2024; Qin and Zhang, 2020; Sipahi et al., 2023). Empirical studies have demonstrated that the activation of NF-κB and the presence of pro-inflammatory cytokines such as IL-1β and TNF-α can enhance the expression and activity of iNOS, promotes the production of nitric oxide (NO), induces the release of glutamate-containing vesicles from astrocytes, inhibits the reuptake of glutamate, and consequently elevates extracellular glutamate concentrations (Ida et al., 2008; Olivenza et al., 2000; Du et al., 2022). Consequently, oxidative stress interacts with inflammation to exacerbate depressive symptoms and *R. glutinosa-Lilium* may exert an antidepressant effect by reducing the levels of pro-inflammatory cytokines IL-1β and TNF-α and modulating the tryptophan-kynurenine pathway (Guo et al., 2024).

**TABLE 6:** The active substances in *Rehmannia glutinosa-Lilium* that regulate inflammation and oxidative stress.

Extracts/metabolites	Controls	Model	Animal/cell	Dose range tested	Duration	Key indicators	References
Geniposide	Fluoxetine hydrochloride (20 mg/mL)	RRS	Male ICR mice	50, 100 mg/kg	15 days	TNF-α↓, IL-1β↓	Zhao et al. (2018)
Ethanol extract of *Lilium*	Mock	LPS	RAW264.7 cells	0–300 μg/mL	24 h	COX-2↓, TNF-α↓, iNOS↓, NF-κB↓	Han et al. (2018)
Quercetin	0.3% carboxymethyl cellulose	CUS	Swiss albino mice	30 mg/kg	26 days	IL-6↓, TNF-α↓, IL-1β↓, COX-2↓	Mehta et al. (2017)
Luteolin	Mock	CUMS	Male Kunming mice	20, 40, 60 mg/kg	21 days	SOD↑, GSH-Px↑, MDA↓	Liu et al. (2013)
Kaempferol	Fluoxetine hydrochloride (10 mg/mL)	CSDS	Male CD1 and C57 mice	10, 20 mg/kg	35 days	IL-6↓, iNOS↓, IL-1β↓, COX-2↓, SOD↑, CAT↑, GSH-Px↑, GST↑, MDA↓	Gao et al. (2019)
Rehmannia glycoside D	Mino (0.1 μmol/L)	LPS	N9 cells	5, 10, 20 μmol/L	24 h	iNOS↓, IL-6↓, IL-1β↓	Wang J et al. (2021)
Catalpol	Fluoxetine hydrochloride (10 mg/mL)	CUMS	Male SD rat	10 mg/kg	35 days	SOD↑, CAT↑, GSH-Px↑, GST↑, GSH↑, MDA↓	Wang Y. L et al. (2021)
	Mitochondrion-targeted antioxidant peptide SS31 (5 mg/kg)	CUMS	Male C57BL/6 mice	20 mg/kg	35 days	IL-1β↓, TNF-α↓, iNOS↓	Wang Y. T. et al. (2021)

**Note:** SOD, superoxide dismutase; MDA, malondialdehyde; GSH-Px, Glutathione peroxidase; CAT, catalase; GST, Glutathione S-transferase; COX-2, Cyclooxygenase-2; TNF-α, Tumor necrosis factor-α; RRS, repeated restraint stress; INOS, inducible nitric oxide synthase; IL-6, Interleukin-6; CSDS, chronic social defeat stress; IL-1β, Interleukin-1β; CUMS, chronic unpredictable mild stimulation; LPS, lipopolysaccharides; CUS, Chronic unpredicate stress.

### 5.4 Effect of active metabolites of *Rehmannia glutinosa-Lilium* on glutamic acid

Neuroimaging and autopsy studies found that depression patients’ plasma, cerebrospinal fluid, and brain glutamate (Glu) concentrations were higher, and serum Glu levels were positively correlated with MDD severity (Zhang et al., 2013; Yan, 2022). With ketamine, a glutamate receptor (NMDAR) antagonist, as a quick-acting antidepressant, the role of glutamatergic nervous system in depression has received widespread attention (Murrough et al., 2017). Glu homeostasis is maintained by the glutamate-glutamine cycle in the central nervous system, and neurons and astrocytes provide a strong guarantee of neuronal activity (Eid et al., 2016; Mahmoud et al., 2019). A high concentration of Glu damages nerve cells and overstimulates glutamate receptors (NMDAR, etc.), which may contribute to depression (Wu et al., 2014). In the rat model of depression induced by CUMS, the levels of NMDAR phosphorylation and subunit NR1/NR2B protein increased significantly, the abnormal concentration of Glu in synaptic space led to the overactivation of extra synaptic NMDAR, and a large amount of Ca^2+^ influx led to intracellular Ca^2+^ overload, resulting in nerve cell death (Li et al., 2018; Beneyto et al., 2007).

**FIGURE 4:**
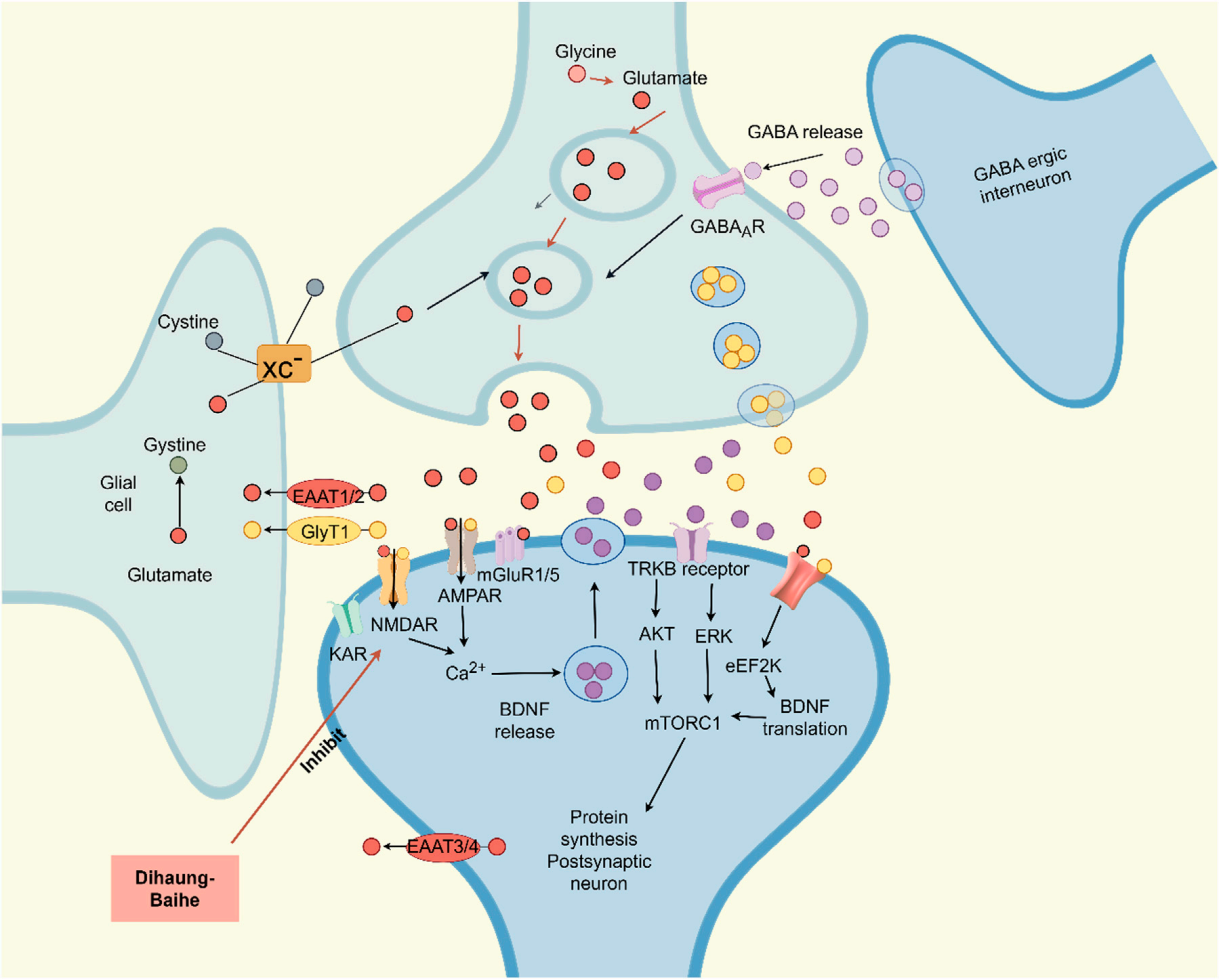
Upon depolarization of presynaptic glutamatergic neurons, gamma-aminobutyric acid (GABA) receptors inhibit the fusion of glutamate-containing vesicles with the presynaptic membrane. Additionally, Group II metabotropic glutamate receptors (mGluRs) modulate glutamate release by inhibiting adenylyl cyclase activity, thereby indirectly influencing synaptic plasticity and long-term potentiation (LTP). In a postsynaptic glutamatergic neuron, the activation of N-methyl-D-aspartate receptors (NMDARs) via brain-derived neurotrophic factor (BDNF) can be associated with the initiation of neurotrophic or apoptotic pathways. Subsequently, BDNF-TrkB signaling enhances the activation of extracellular signal-regulated kinase (ERK),serine/threonine-specific protein kinase (Akt), and the mechanistic target of rapamycin complex 1 (mTORC1) signaling pathways.

Modern studies have found that catalpol in *R. glutinosa* can significantly reverse the decrease of cell survival rate and muscarinic receptor density induced by L-Glu, suggesting that catalpol may have neuroprotective effects by regulating the cholinergic nervous system (Wang et al., 2008). Acubin inhibits glutamate receptor NMDAR1 and oxidative stress, thereby improving Glu excitotoxicity, and improving PC-12 cell damage induced by Glu, which has potential activity in the treatment of depression (Lu et al., 2022). The phenylethanol glycoside compound echinacoside exhibits neuroprotective, antiinflammatory, antioxidant, antiviral, cardiac activity, and many other biological properties (Liu et al., 2018). Moreover, echinacoside crosses the blood-brain barrier, suggesting potential clinical application for neurological diseases (Zhu et al., 2013). Echinacea glycoside may improve Glu-induced PC-12 cell damage by reducing intracellular Ca^2+^ accumulation, inhibiting NMDAR1 protein expression and antioxidation (Lu et al., 2021). The consumption of berberine significantly decreased ROS production, lipid peroxidation, and DNA fragmentation in glutamate-damaged hippocampal cells, increasing glutathione content and SOD activity, and the anti-apoptotic effect of berberine was demonstrated by reducing the overexpression of Caspase-3 and Bax/Bcl-2 induced by glutamate (Xue, 2021) (Figure 4; Table 7). *In vivo*, elevated glutamate levels lead to the over-activation of NMDA receptors, causing a substantial influx of Ca2+ and subsequent Ca2+ overload (Zhou et al., 2024). This cascade triggers downstream pathways that result in the production of reactive oxygen species and mitochondrial dysfunction, ultimately leading to neuronal damage (Wang Y. L et al., 2021). *Rehmannia glutinosa-Lilium* may exert an antidepressant effect by mitigating Ca2+ accumulation within cells and inhibiting the expression of glutamate receptors.

**TABLE 7:** Active substances regulating glutamate in *Rehmannia glutinosa-Lilium.*

Extracts/metabolites	Controls	Model	Animal/cell	Dose range tested	Duration	Key indicators	References
Catalpol	Mock	L-Glu	PC-12	1, 10, 100 μmol/L	23 h	M receptor↑	Wang et al. (2008)
Aucubin	Fluoxetine (0.3 μmol/L)	GLU	PC-12 cells	1, 5, 10, 20, 40 μmol/L	24 h	ROS↓, SOD↑, NMDAR1↓, LDH↓	Lu et al. (2022)
Echinacoside glycoside	Fluoxetine (0.3 μmol/L)	GLU	PC-12 cells	2, 5, 10 μmol/L	24 h	ROS↓, SOD↑, NMDAR1↓, LDH	Lu et al. (2021)
Berberine	0.5% carboxymethyl cellulose	STZ	Male ICR mice	50 mg/kg	42 days	ROS↓, SOD↑, Caspase-3↓, Bax/Bcl-2↓	Xue (2021)

**Note:** SOD, superoxide dismutase; ROS, reactive oxygen species; NMDAR, N-methyl-D-aspartate receptor; LDH, lactate dehydrogenase; Glu, Glutamate.

### 5.5 The hypothalamus-pituitary-adrenal (HPA) axis is influenced by active metabolites of *Rehmannia glutinosa-Lilium*

Depression can be caused by excessive excitation of the HPA neurohormone, which regulates stress states in the body (Juruena et al., 2018; Tang et al., 2019). The hypothalamus begins with adrenocorticotropic hormone-releasing hormone (CRH) secreted by the paraventricular nucleus and then stimulates the pituitary gland, where the anterior lobe releases adrenocorticotropic hormone (ACTH), which in turn induces adrenal gland secretion of CORT. In turn, CORT regulates the stress response by reducing its own secretion by sending feedback signals to the hypothalamus and pituitary to reduce the production of CRH and ACTH (Sarno et al., 2021; Plotsky et al., 1998). It was found that the secretion and response of CORT, the level of CRH in cerebrospinal fluid and inflammation increased in patients with severe depression (Zunszain et al., 2011; Amasi-Hartoonian et al., 2022; Horowitz et al., 2020). The increase of CORT level caused by imbalance of HPA axis was directly related to depressive symptoms (Wang et al., 2022). Behavioral test results of mice after injection of CORT suggested depression-like behavior, and serum levels of CORT, ACTH, and CRH increased dose-dependently and over time (Chen et al., 2021; Mikulska et al., 2021; Lok et al., 2012; Joseph and Golden, 2017).

**FIGURE 5:**
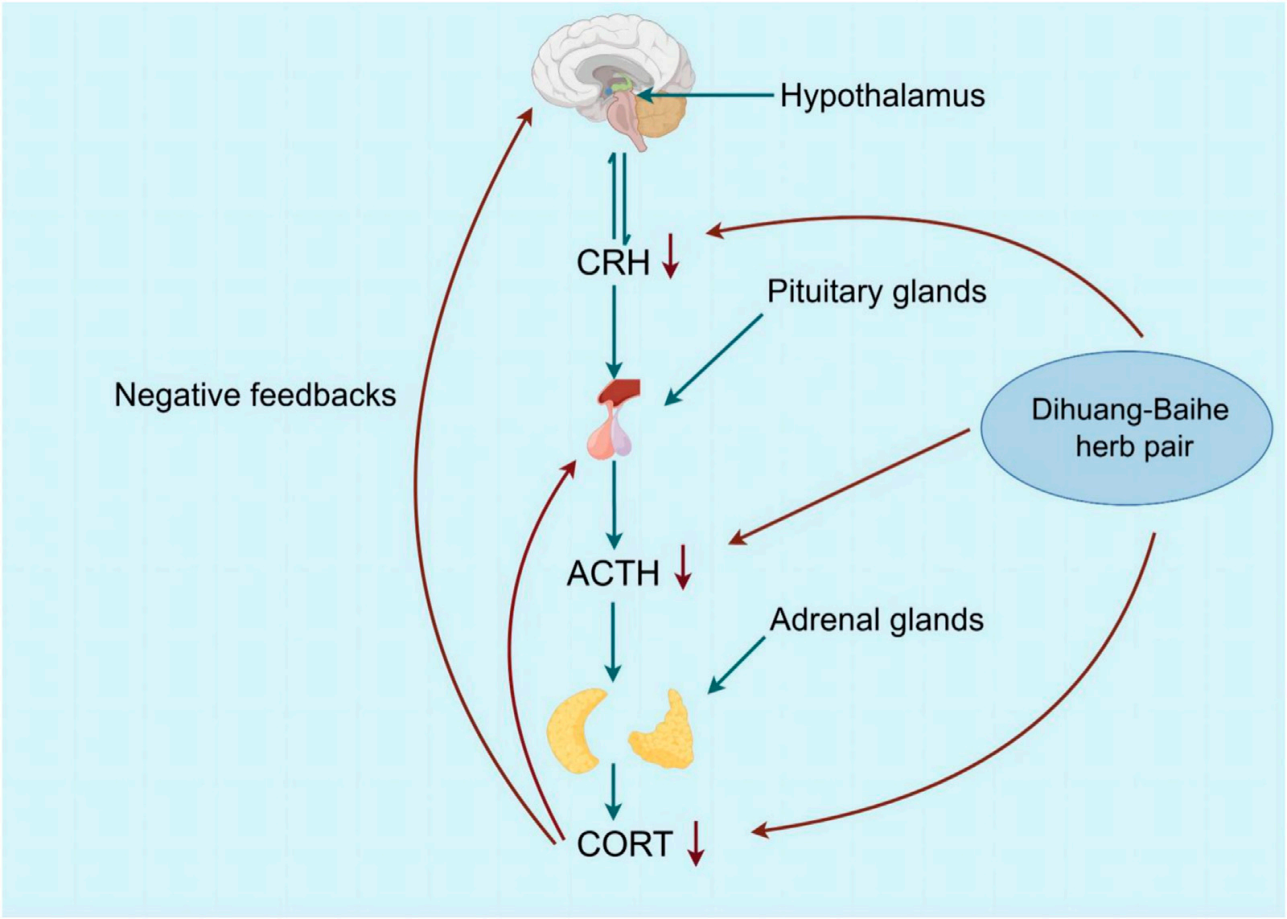
The active metabolites of *Rehmannia glutinosa-Lilium* can reduces COR levels and inhibits further release of ACTH and CRH, and depressive symptoms disappear.

Geneniposide was found to restore the negative feedback between the CRH expression and HPA axis injured by CUMS, which inhibited its high activity, and a significant reduction in CORT serum levels, as well as CRH mRNA expression, was also observed, but ACTH levels were not significantly affected (Cai et al., 2015). *Lilium* saponins have been found to exhibit an antidepressant effect by suppressing the hyperactivity of the HPA axis, leading to a reduction in circulating levels of COR, ACTH and CRF mRNA in rats (Guo et al., 2010). In mice, the antidepressant effect of catalpol is attributed to its ability to regulate both NF-κB and Nrf2, thereby inhibiting HPA axis hyperactivity, central inflammation, oxidative damage, and depression-like behavior induced by CORT (Song et al., 2021). Berberine has the potential to induce a calming and hypnotic effect through the inhibition of the HPA axis and the augmentation of the levels of 5-HT and NE in the hypothalamus of a mouse model of insomnia induced by PCPA (Zhou et al., 2014) (Figure 5; Table 8). When 5-HT levels in the brain are low, ACTH secretion of the pituitary gland increases, resulting in increased secretion of peripheral cortisol, which suggest that the 5-HT system in the brain exerts an inhibitory effect on ACTH secretion (Zhao et al., 2021a). Following the interaction between cortisol and glucocorticoid receptors, there is an activation of tyrosine aminotransferase and tryptophan pyrroliase, which reduces the synthesis of the 5-HT and NE precursors, tyrosine and tryptophan, resulting in a decrease in the content of monoamine transmitters in the brain and worsening anxiety and depression (Arborelius and Eklund, 2007; Greenstein and Hunt, 2023). Therefore, *R. glutinosa-Lilium* regulates the HPA axis by reducing the content of hormones such as CORT, ACTH and CRH, thus playing an antidepressant role.

**TABLE 8:** Active substances regulating hypothalamus-pituitary-adrenal axis in *Rehmannia glutinosa-Lilium*.

Extracts/metabolites	Controls	Model	Animal/cell	Dose range tested	Duration	Key indicators	References
Geniposide	Fluoxetine hydrochloride (10 mg/mL)	CUMS	Male SD rats	20, 50, 100 mg/kg	21 days	CORT↓, ACTH↓, CRH mRNA↓	Cai et al. (2015)
*Lilium* saponins	Fluoxetine hydrochloride (2 mg/mL)	CUMS	Male SD rats	12, 24, 48 mg/kg	21 days	CORT↓, ACTH↓, DA↑, 5-HT↑	Guo et al. (2010)
Catalpol	Fluoxetine hydrochloride (20 mg/mL	CORT	Male Kunming mice	20 mg/kg	35 days	Nrf2↑, NF-κB↓, IL-1β↓, TNF-α↓, iNOS↓, NO↓, GSH-Px↑, GST↑, SOD↑, MDA↓	Song et al. (2021)
Berberine	Diazepam (2.25 mg/kg)	PCPA	Female Kunming mice	75 mg/kg	5 days	NE↑, 5-HT↑, HPA↓	Zhou et al. (2014)

**Note:** CORT, cortisol; ATCH, adrenocorticotropin; DA, dopamine; 5-HT, 5-hydroxytryptamine; NF-κB, Nuclear factor kappa-B; iNOS, inducible nitric oxide synthase; Nrf2, Nuclear factor E2-related factor 2; IL-1β, Interleukin-1β; NO, nitric oxide; TNF- α, Tumor necrosis factor-α; GSH-Px, Glutathione peroxidase; SOD, superoxide dismutase; GST, Glutathione S-transferase; CUMS, chronic unpredictable mild stimulation; MDA, Malondialdehyde.

### 5.6 Effects of active metabolites of *Rehmannia glutinosa-Lilium* on intestinal microorganisms

The gut-brain axis, which refers to the complex interaction between the gastrointestinal tract and the brain, has been demonstrated to exert a significant impact on emotional regulation, cognitive processes, and the central nervous system, ultimately contributing to the pathogenesis of depression (Tian et al., 2022; Kim et al., 2023; Xie et al., 2023). Recent research has demonstrated notable alterations in the intestinal microbiota of both depressed individuals (Huang Y et al., 2023) and animal models of depression (Xie et al., 2023), indicating a strong association between gut flora and depression (Zhang M. et al., 2021; Zhao N. et al., 2023). By regulating coding RNA, non-coding RNA and various signal pathways, intestinal flora can regulate not only the function of hippocampal and microglia, but also the expression level of BDNF and immune inflammatory response related to depression, which ultimately affect depression’s occurrence and development, suggesting that a potential target for treating depression could be inhibition of intestinal flora (Chen et al., 2022; Xu M. et al., 2023).

**FIGURE 6:**
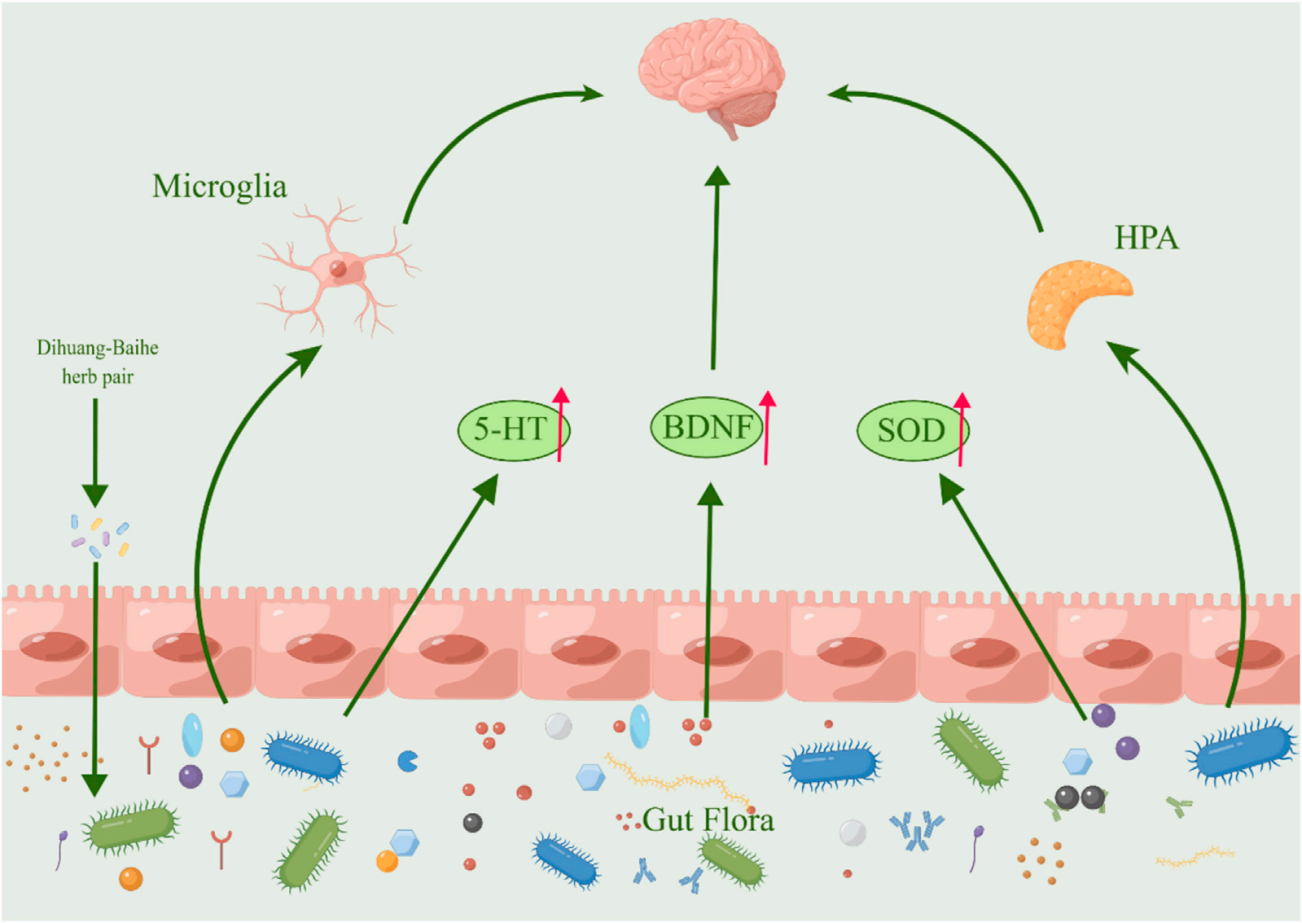
The active metabolites in *Rehmannia glutinosa-Lilium* can regulate intestinal microorganisms. (HPA, Hypothalamus-pituitary-adrenal; 5-HT, 5-hydroxytryptamine; BDNF, Brain-derived neurotrophic factor).

The anti-fatigue, antidepressant, antibacterial, and other effects of *Lilium* polysaccharides have been demonstrated in modern pharmacological studies (Gao et al., 2015). *Lilium* polysaccharides regulate intestinal flora imbalance by inhibiting the increase of LPS, IL-6, and TNF- α, increasing the content of secretory immunoglobulin A (SIgA) and regulating intestinal flora imbalance by cultivating beneficial bacteria and inhibiting harmful bacteria (Zhao et al., 2020). In STZ-induced diabetic mice, oral administration of 300 mg/kg *R. glutinosa* stachyose can significantly lower blood glucose levels, restore the number of *Lactobacillus* and some normal bacteria reduced by disease to a certain extent, which has the dual effect of regulating blood sugar and intestinal flora (Wang, 2013). Following the administration of kaempferol, the intestinal microbiota of mice with ulcerative colitis exhibited an increase in richness and the relative ratio of Firmicutes and *Bacteroides* was observed to increase, while the relative abundance of pathogenic species decreased and the abundance of probiotics increased (Qu, 2021). Berberine can significantly reduce the levels of both Trichobacterium and *Clostridium* diffrium in rats, increase the levels of Rumen and Lactic Acid Bacteria, and inhibit pro-inflammatory cytokines, thus inhibiting the overactivated inflammatory response by regulating rat intestinal flora (Huang D. X. et al., 2023). In mice with non-alcoholic fatty liver disease (NAFLD), the administration of chlorogenic acid resulted in an increase in intestinal flora and the secretion of glucagon-like peptide-1 (GLP-1), which is known to regulate inflammation (Shi et al., 2021) (Figure 6; Table 9). Turicibacter is an intestinal bacterium that expresses a sodium transporter-related protein exhibiting sequence and structural homology to mammalian neurotransmitters, among which DA, NE, acetylcholine, and GABA are all neurotransmitters closely associated with depression, thus causing depressive mood and behavior (Fung et al., 2019; Feng et al., 2024). We hypothesize that *R. glutinosa-Lilium* may influence the synthesis and transport of neurotransmitters by effectively preventing intestinal microorganisms from entering systemic circulation, inhibiting the body’s immune response, and regulating the abundance of intestinal flora, thereby achieving therapeutic effects in the treatment of depression.

**TABLE 9:** Active substances regulating intestinal microorganisms in *Rehmannia glutinosa-Lilium.*

Extracts/metabolites	Controls	Model	Animal/cell	Dose range tested	Duration	Key indicators	References
*Lilium* polysaccharides	lizhu intestine (10 mg/kg)	Lincomycin hydrochloride	Male Kunming mice	50, 100, 200 mg/kg	21 days	LPS↓, IL-6↓, TNF-α↓	Zhao et al. (2020)
*Rehmannia glutinosa* stachyose	Metformin (400 mg/kg)	STZ	Male Kunming mice	300 mg/kg	49 days	*Lactobacillus*↓, *Bacteroides*↓	Wang (2013)
Kaempferol	Mock	UC	Female C57BL/6J mice	50 mg/kg	14 days	Firmicutes/*Bacteroides*↑, Proteobacteria↓	Qu (2021)
Berberine	Mock	NAFLD	SD rat	150 mg/kg	112 days	*Clostridium* ↓, Lactic acid bacteria↑, IL-6↓, TNF-α↓	Huang D. X. et al. (2023)
Chlorogenic acid	Mock	NAFLD	Male C57BL/6 mice	60 mg/kg	84 days	GLP-1↑, *Escherichia coli*↓, IL-6↓, TNF-α↓	Shi et al. (2021)

**Note:** LPS, lipopolysaccharide; IL-6, Interleukin-6; TNF-α, Tumor necrosis factor-α; STZ, streptozotocin; UC, Ulcerative colitis.

## 6 Toxicity studies

The extract of *Rehmannia* was evaluated for acute toxicity in mice using the LD50 and MTD methods, with no fatalities observed. Concurrently, a subchronic toxicity study was conducted on 80 Sprague-Dawley rats, which were allocated into four groups: low (1,670 mg/kg), medium (8,330 mg/kg), high (16,700 mg/kg) doses of *Rehmannia* extract, and a control group. After 30 days of continuous intragastrical administration of the extract, there were results no significant differences in body weight, blood biochemical parameters, organ coefficients, or visceral histopathology between the treatment groups and the control group (Liu J et al., 2017). According to the acute toxicity classification standard of the World Health Organization (WHO) and the results from both acute and subchronic toxicity tests, *Rehmannia* extract is deemed safe and non-toxic at clinical doses. The cytotoxicity of the water extract of *Lilium*, both before and after sulfur fumigation, was assessed. The cytotoxicity of the aqueous extract of *Lilium*, both prior to and following sulfur fumigation, was assessed. Concentrations ranging from 0 to 800 mg/L demonstrated that the post-fumigation aqueous extract of *Lilium* exhibited no significant impact on the viability of human liver LO2 cells, human renal proximal tubule HK-2 cells, and rat adrenal pheochromocytoma PC-12 cells. Furthermore, no significant differences were observed when compared to the pre-fumigation aqueous extract. These findings suggest that *Lilium* concentrations between 0 and 800 mg/L do not induce cytotoxic effects (Zhang et al., 2023). *Rehmannia* and *Lilium* are not only recognized in the Chinese Pharmacopoeia, but are also listed among Chinese medicinal materials utilized for both therapeutic and dietary purposes, with a long-standing history of consumption. However, certain metabolites within *Rehmannia* and *Lilium* may exhibit toxic side effects.

Although colchicine in lilies is relatively less toxic, its metabolism in the liver through deacetylation results in the formation of the more toxic compound dicolchicine. This metabolite repeatedly interacts with the gastrointestinal mucosa during enterohepatic circulation, leading to symptoms of poisoning such as nausea, vomiting, and abdominal pain. These interactions can further result in damage to liver and kidney function and may lead to metabolic acidosis, as well as respiratory and circulatory failure (Liu et al., 2024). Furthermore, colchicine exhibits significant cardiotoxicity, with severe cases potentially resulting in mortality due to circulatory failure and fatal arrhythmias (Mullins et al., 2000). Liver biopsy specimens from mice treated with AU did not reveal any abnormal histological findings. Following a single intraperitoneal injection of 1–100 mg/kg AU, all Wistar rats survived, but administration of 100 mg/kg AU led to paralysis (Xue et al., 2012). Acute toxicity assessments conducted on mice with gavage doses of 10, 20, and 40 g/kg AU indicated that mice receiving 40 g/kg AU experienced a slight reduction in free movement and food intake, along with the presence of fatty or soft stools. Nevertheless, these phenomena gradually normalized by days 2–3 and no animals exhibited symptoms of poisoning or mortality within 14 days post-treatment (Li, 2011). Consequently, while medicinal and edible plants are generally considered safe, they are not devoid of potential adverse effects, including side effects and toxicity, which may be dose-dependent, particularly in long-term studies.

## 7 Conclusion and future perspectives

Globally, depression affects hundreds of millions of people, but because depression affects many systems of the body, the treatment of depression is a difficult problem for both modern medicine and TCM. The treatment of depression is currently limited to a single target or a single signal pathway, target-signal pathway interactions are not sufficiently discussed in depth, and the drugs used in clinics still cannot fully cure a variety of depression-related diseases.

The pathogenesis of depression is interconnected, with no single factor acting independently. For instance, an imbalance in monoamine neurotransmitters can lead to increased inflammation, while the inflammatory response can exacerbate the reduction of 5-HT levels, collectively contributing to the development of depression (Guo et al., 2024). Glutamic acid can elevate NO levels, and NO, in turn, can regulate the release of neurotransmitters such as 5-HT and DA (Wang R. et al., 2023). The hyperactivation of the HPA axis and the subsequent excessive secretion of corticosterone lead to the compromise of the blood-brain barrier, which in turn results in neuronal damage and contributes to the pathophysiology of depression (Zhao et al., 2021b). Additionally, the gut microbiota plays a significant role in modulating depressive states by influencing inflammatory pathways and altering the synthesis of neurotransmitters (Feng et al., 2024). Combinations of TCM compounds have multiple advantages, such as multi-metabolites, multi-pathway, and multi-target treatment. Chinese traditional medicine’s active metabolites is a monomer compound extracted and purified from TCM, which is the TCM’s main metabolites and its compound preparations to exert its pharmacological effects, and its target, signaling pathway and mechanism for treating diseases are relatively clear (Xing et al., 2024c). In this review, we reviewed for the first time that *R. glutinosa-Lilium* has an active ingredient in antidepressant. We found that catalpol, geniposide, *Lilium* saponins, gallic acid and berberine can relieve depression by enhancing the levels of monoamine neurotransmitters in the brain, such as 5-HT, DA, and NE. Catalpol, Regaloside A, Rhmannioside D and chlorogenic acid on depression can be attributable to its ability to upregulate the expression of BDNF and TrkB receptors. Geniposide, ethanol extract of *Lilium*, quercetin, luteolin, kaempferol, Rhmannioside D and catalpol inhibit the occurrence of depression by improving oxidative stress and inflammation. Catalpol, aucubin, echinacosid and berberine reduces depression symptoms by reducing Glu levels and NMDAR expression. Geniposide, *Lilium* Saponins, catalpol and berberine inhibits the release of hormones such as ACTH and CRH, thereby reducing depression symptoms. *Lilium* Polysaccharides, stachyose, kaempferol, berberine and gallic acid regulates intestinal flora by inhibiting harmful bacteria, therefore reducing depression symptoms.

According to this review, we found that catalpol, acteoside, gallic acid, berberine, Regaloside A, chlorogenic acid, Rhmannioside D, geniposide, quercetin, luteolin, kaempferol, aucubin, echinacoside, stachyose and other main active metabolites were found in the *R. glutinosa-Lilium* (Table 10). Although the therapeutic effect of *R. glutinosa-Lilium* on depression has been substantiated, its application in the development of antidepressant agents remains relatively underexplored. Active metabolites such as catalpol, ralinosin A, and genipine present significant potential for development as lead compounds to enhance pharmacological efficacy, which provides a reliable basis for the development of antidepressant drugs. Secondly, leveraging the traditional prescription of Baihe Dihuang Decoction, advanced methodologies such as network pharmacology, bioinformatics, and systems biology were employed to optimize the formulation, enhance therapeutic efficacy, and refine the compatibility, which aim to harness the multi-metabolites and multi-target treatment characteristics inherent in traditional Chinese medicine, thereby augmenting its antidepressant effects. Therefore, the future research direction should focus on using new technology to systematically describe the antidepressant tool of TCM from many aspects, multi-targets, and multi-levels, and simultaneously explore new antidepressant targets and develop fast, effective, and specific antidepressant drugs to provide a new direction for clinical trials of depression.

**TABLE 10:** The main antidepressant active metabolites in *Rehmannia glutinosa-Lilium*.

Metabolites	Structural formula	Source	References
Catalpol	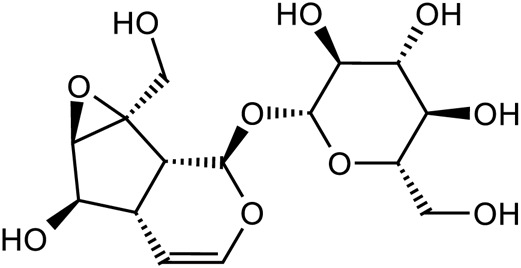	*Rehmannia glutinosa*	Wang et al. (2014), Wang Y. L et al. (2021), Wang et al. (2008), Wang Y. T. et al. (2021), and Song et al. (2021)
Acteoside	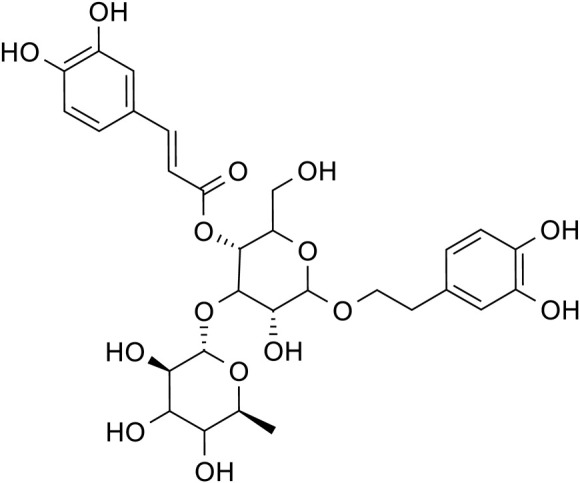	*Rehmannia glutinosa*	Xue X. et al. (2022)
Gallic acid	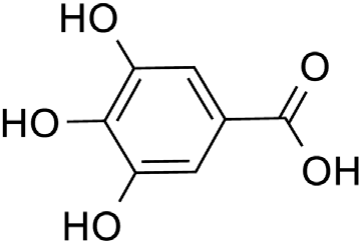	*Lilium*	Can et al. (2017)
Berberine	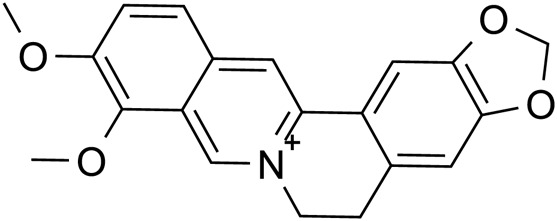	*Lilium*	Peng et al. (2007), Xue (2021), Zhou et al. (2014), and Huang Y et al. (2023)
Regaloside A	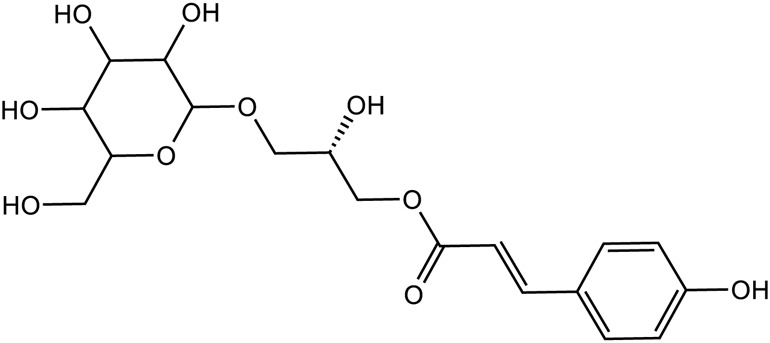	*Lilium*	Yuan et al. (2021)
Chlorogenic acid	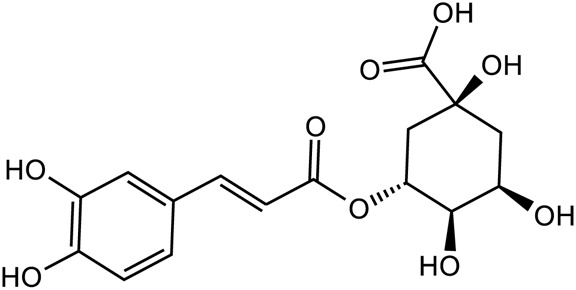	*Lilium*	Liu et al. (2021) and Shi et al. (2021)
Rhmannioside D	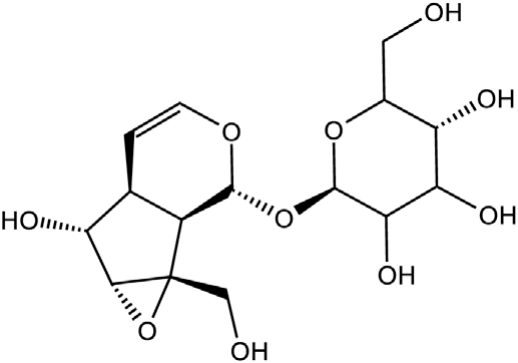	*Rehmannia glutinosa*	Zhang et al. (2022) and Wang Y. L et al. (2021)
Geniposide	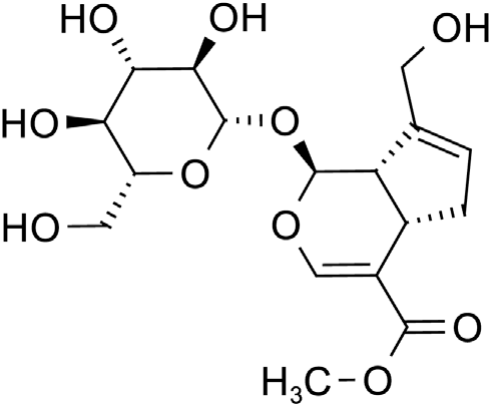	*Rehmannia glutinosa*	Zhao et al. (2018) and Cai et al. (2015)
Quercetin	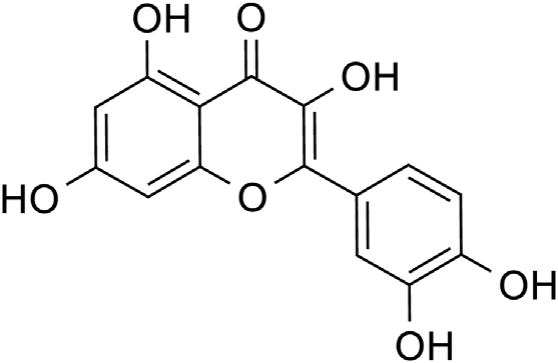	*Lilium*	Mehta et al. (2017)
Luteolin	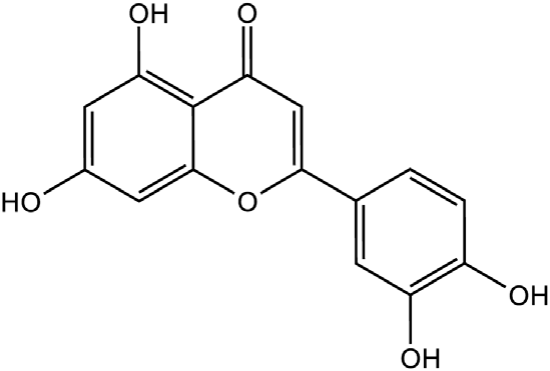	*Lilium*	Liu et al. (2013)
Kaempferol	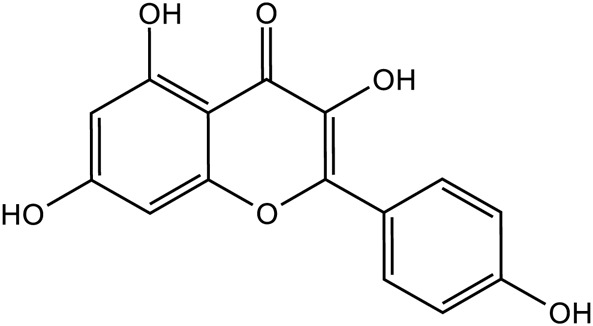	*Lilium*	Gao et al. (2019) and Qu (2021)
Aucubin	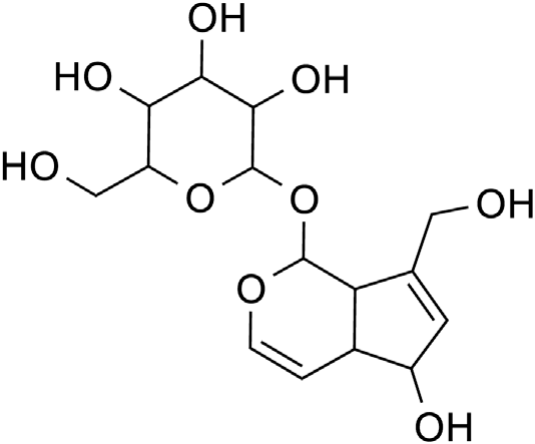	*Rehmannia glutinosa*	Lu et al. (2022)
Echinacoside	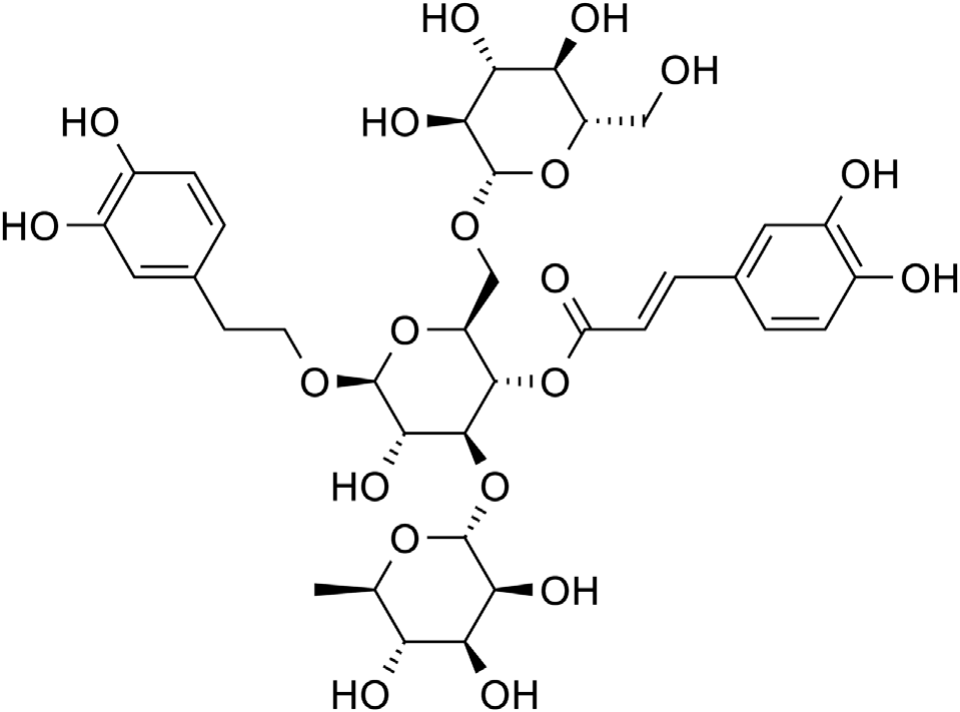	*Rehmannia glutinosa*	Lu et al. (2021)
Lupeose	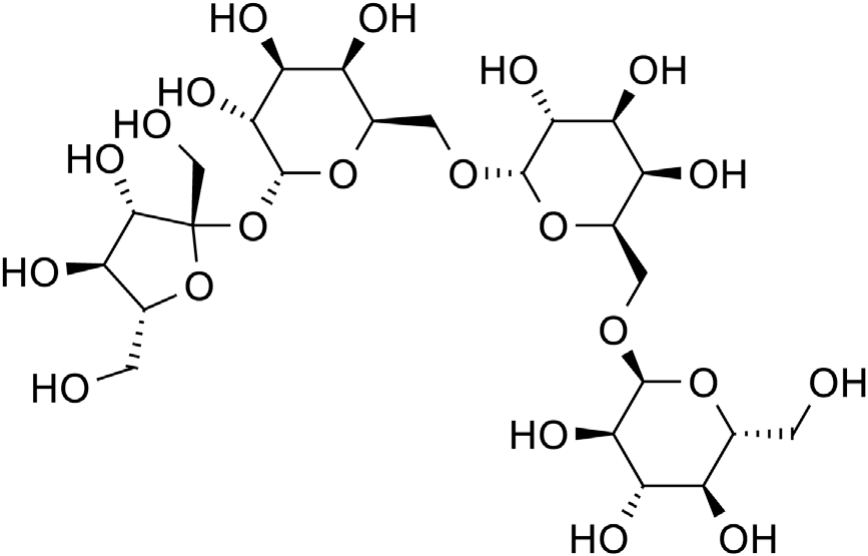	*Rehmannia glutinosa*	Wang (2013)

## References

[B1] Amasi-Hartoonian N. Sforzini L. Cattaneo A. Pariante C. M. (2022). Cause or consequence? Understanding the role of cortisol in the increased inflammation observed in depression. Curr. Opin. Endocr. Metab. Res. 24, 100356. 10.1016/j.coemr.2022.100356 35634363 PMC7612780

[B2] Arborelius L. Eklund M. B. (2007). Both long and brief maternal separation produces persistent changes in tissue levels of brain monoamines in middle-aged female rats. Neuroscience 145 (2), 738–750. 10.1016/j.neuroscience.2006.12.007 17222517

[B3] Beneyto M. Kristiansen L. V. Oni-Orisan A. Mccullumsmith R. E. Meador-Woodruff J. H. (2007). Abnormal glutamate receptor expression in the medial temporal lobe in schizophrenia and mood disorders. Neuropsychopharmacology 32 (9), 1888–1902. 10.1038/sj.npp.1301312 17299517

[B4] Beurel E. Toups M. Nemeroff C. B. (2020). The bidirectional relationship of depression and inflammation: double trouble. Neuron 107 (2), 234–256. 10.1016/j.neuron.2020.06.002 32553197 PMC7381373

[B5] Bhatt S. Nagappa A. N. Patil C. R. (2020). Role of oxidative stress in depression. Drug Discov. Today. 25 (7), 1270–1276. 10.1016/j.drudis.2020.05.001 32404275

[B6] Blomqvist S. Hognas R. S. Virtanen M. Lamontagne A. D. Magnusson H. L. (2023). Job loss and job instability during the COVID-19 pandemic and the risk of depression and anxiety among Swedish employees. Health 22, 101424. 10.1016/j.ssmph.2023.101424 PMC10158169 37159634

[B7] Boucas A. P. Rheinheimer J. Lagopoulos J. (2022). Why severe COVID-19 patients are at greater risk of developing depression: a molecular perspective. Neuroscientist 28 (1), 11–19. 10.1177/1073858420967892 33135582

[B8] Brites D. Fernandes A. (2015). Neuroinflammation and depression: microglia activation, extracellular microvesicles and microRNA dysregulation. Front. Cell. Neurosci. 9, 476. 10.3389/fncel.2015.00476 26733805 PMC4681811

[B9] Buttiker P. Weissenberger S. Esch T. Anders M. Raboch J. Ptacek R. (2022). Dysfunctional mitochondrial processes contribute to energy perturbations in the brain and neuropsychiatric symptoms. Front. Pharmacol. 13, 1095923. 10.3389/fphar.2022.1095923 36686690 PMC9849387

[B10] Cai L. Li R. Tang W. J. Meng G. Hu X. Y. Wu T. N. (2015). Antidepressant-like effect of geniposide on chronic unpredictable mild stress-induced depressive rats by regulating the hypothalamus-pituitary-adrenal axis. Eur. Neuropsychopharmacol. 25 (8), 1332–1341. 10.1016/j.euroneuro.2015.04.009 25914157

[B11] Can O. D. Turan N. Demir O. U. Ozturk Y. (2017). Antidepressant-like effect of gallic acid in mice: dual involvement of serotonergic and catecholaminergic systems. Life Sci. 190, 110–117. 10.1016/j.lfs.2017.09.023 28942286

[B12] Chen H. Ye C. Wu C. Zhang J. Xu L. Wang X. (2023). Berberine inhibits high fat diet-associated colorectal cancer through modulation of the gut microbiota-mediated lysophosphatidylcholine. Int. J. Biol. Sci. 19 (7), 2097–2113. 10.7150/ijbs.81824 37151876 PMC10158016

[B13] Chen L. Wang X. Zhang Y. Zhong H. Wang C. Gao P. (2021). Daidzein alleviates hypothalamic-pituitary-adrenal Axis hyperactivity, ameliorates depression-like behavior, and partly rectifies circulating cytokine imbalance in two rodent models of depression. Front. Behav. Neurosci. 15, 671864. 10.3389/fnbeh.2021.671864 34733143 PMC8559531

[B14] Chen W. Chen Y. X. (1963). Jinkui Fang GE Kuo. Shanghai: Shanghai Sci and Techno Press.

[B15] Chen W. L. Yan X. R. Gao J. P. Song G. H. (2022). Research progress on the regulatory mechanism of intestinal flora in the occurrence of depression. Chin. J. Comp. Med. 32 (10), 130–135. 10.3969/j.issn.1671-7856.2022.10.017

[B16] Chen Y. Xing Z. Chen J. Sun C. Liu Y. Peng C. (2024). SIRT1 activation by Ligustrazine ameliorates migraine via the paracrine interaction of microglia and neurons. Phytomedicine 135, 156069. 10.1016/j.phymed.2024.156069 39341123

[B17] Chi X. Wang S. Baloch Z. Zhang H. Li X. Zhang Z. (2019). Research progress on classical traditional Chinese medicine formula Lily Bulb and Rehmannia Decoction in the treatment of depression. Biomed. Pharmacother. 112, 108616. 10.1016/j.biopha.2019.108616 30780102

[B18] Da M. J. (1987). Progenitors. Beijing: People's Health Publishing House.

[B19] Deng L. Zhou X. Tao G. Hao W. Wang L. Lan Z. (2022). Ferulic acid and feruloylated oligosaccharides alleviate anxiety and depression symptom via regulating gut microbiome and microbial metabolism. Food Res. Int. 162 (Pt A), 111887. 10.1016/j.foodres.2022.111887 36461269

[B20] Du Q. Chen L. He W. Lao J. Cai Y. Huang J. (2022). Effects of Polygonum polygonum polysaccharide on the activity of RAW264.7 cells and expression of inflammatory factors TNF-α, IL-6 and iNOS. Chin. Tradit. Pat. Med. 44 (08), 2676–2679. 10.3969/j.issn.1001-1528.2022.08047

[B21] Du Y. M. Zhang Y. Q. Wang Z. Q. Min X. Ya Y. L. Wang Y. F. (2023). Characteristics of somatic symptoms and their correlations with brain-derived neurotrophic factor and inflammatory cytokinesin patients with major depressive disorder. Chin. Gen. Pract. 26 (12), 1463–1471. 10.12114/j.issn.1007-9572.2022.0652

[B22] Duman R. S. Monteggia L. M. (2006). A neurotrophic model for stress-related mood disorders. Biol. Psychiatry. 59 (12), 1116–1127. 10.1016/j.biopsych.2006.02.013 16631126

[B23] Eid T. Gruenbaum S. E. Dhaher R. Lee T. W. Zhou Y. Danbolt N. C. (2016). The glutamate-glutamine cycle in epilepsy. Adv. Neurobiol. 13, 351–400. 10.1007/978-3-319-45096-4_14 27885637

[B24] El M. M. Guiard B. P. Chernoloz O. Ghanbari R. Katz N. Blier P. (2010). Relevance of norepinephrine-dopamine interactions in the treatment of major depressive disorder. CNS Neurosci. Ther. 16 (3), e1–e17. 10.1111/j.1755-5949.2010.00146.x 20406250 PMC2904493

[B25] Fan Q. Liu Y. Sheng L. Lv S. Yang L. Zhang Z. (2023). Chaihu-Shugan-San inhibits neuroinflammation in the treatment of post-stroke depression through the JAK/STAT3-GSK3β/PTEN/Akt pathway. Biomed. Pharmacother. 160, 114385. 10.1016/j.biopha.2023.114385 36774722

[B26] Feng X. Liu B. Liu Y. Wang D. Liu L. Zhu L. (2024). Effects of Baihe Dihuang decoction on intestinal flora of depressed rats. World. Chin. Med. 19 (06), 780–787. 10.3969/j.issn.1673-7202.2024.06.004

[B27] Filatova E. V. Shadrina M. I. Slominsky P. A. (2021). Major depression: one brain, one disease, one set of intertwined processes. Cells 10 (6), 1283. 10.3390/cells10061283 34064233 PMC8224372

[B28] Fries G. R. Saldana V. A. Finnstein J. Rein T. (2023). Molecular pathways of major depressive disorder converge on the synapse. Mol. Psychiatry. 28 (1), 284–297. 10.1038/s41380-022-01806-1 36203007 PMC9540059

[B29] Fung T. C. Vuong H. E. Luna C. Pronovost G. N. Aleksandrova A. A. Riley N. G. (2019). Intestinal serotonin and fluoxetine exposure modulate bacterial colonization in the gut. Nat. Microbiol. 4 (12), 2064–2073. 10.1038/s41564-019-0540-4 31477894 PMC6879823

[B30] Gao J. Zhang T. Jin Z. Y. Xu X. M. Wang J. H. Zha X. Q. (2015). Structural characterisation, physicochemical properties and antioxidant activity of polysaccharide from Lilium lancifolium Thunb. Food Chem. 169, 430–438. 10.1016/j.foodchem.2014.08.016 25236248

[B31] Gao W. Wang W. Peng Y. Deng Z. (2019). Antidepressive effects of kaempferol mediated by reduction of oxidative stress, proinflammatory cytokines and up-regulation of AKT/β-catenin cascade. Metab. Brain Dis. 34 (2), 485–494. 10.1007/s11011-019-0389-5 30762138

[B32] Gao W. Z. Shen Z. N. (2008). Prescriptions worth thousand gold for emergencies. Beijing: Huaxia publishing house.

[B33] Ge N. Yan G. L. Sun H. Wang X. J. (2023). Research progress on effective constituents in Radix Rehmanniae praeparata. Chin. Herb. Med. 54 (01), 292–302. 10.7501/j.issn.0253-2670.2023.01.031

[B34] Geng X. T. (2022). Research progress on chemical constituents and pharmacological effects of Rehmannia glutinosa. Heilongjiang Sci. 13 (24), 51–53.

[B35] Greenstein A. E. Hunt H. J. (2023). The glucocorticoid receptor modulator relacorilant reverses the immunosuppressive effects of cortisol. Int. Immunopharmacol. 120, 110312. 10.1016/j.intimp.2023.110312 37230031

[B36] Gu H. P. (2005). Required readings for medical professionals. Tradit. Chin. Med. Press.

[B37] Guo D. Fan W. Wang S. Mao Q. Zhang H. Ma K. (2024). Research developmenton the interaction between neuroimmunity and inflammationin the treatment of depressive disorder by traditional Chinese medicine. Chin. J. Comp. Med. 34 (08), 167–177. 10.3969/j.issn.1671-7856.2024.08.019

[B38] Guo Q. P. Gao Y. Li W. M. (2009). Effects of effective parts of Lily on monoamine neurotransmitters in the brain of depression model rats. Chin. Tradit. Pat. Med. 31 (11), 1669–1672.

[B39] Guo Q. P. Gao Y. Li W. M. (2010). Effect of lily saponins on HPA axis in depression model rats. Chin. Pharmacol. Bull. 26 (05), 699–700.

[B40] Han S. Y. Yi Y. S. Jeong S. G. Hong Y. H. Choi K. J. Hossain M. A. (2018). Ethanol extract of Lilium bulbs plays an anti-inflammatory role by targeting the IKK [formula: see text]/[formula: see text]-mediated NF- [formula: see text] B pathway in macrophages. Am. J. Chin. Med. 46 (6), 1281–1296. 10.1142/S0192415X18500672 30149753

[B41] He D. Zhang H. C. Li S. H. Zhou X. Zhang S. H. (2022). Research progress on chemical constituents and pharmacological effects of Baihe (Lilii bulbus) and predictive analysis on quality markers. Chin. Arch. Tradit. Chin. Med. 40 (12), 205–212+303. 10.13193/j.issn.1673-7717.2022.12.043

[B42] He R. He R. P. (2005). Jinkui yaolue. Beijing: People's Health Publishing House.

[B43] He X. Zhang R. Li Z. Yao Z. Xie X. Bai R. (2022). Sini powder with paroxetine ameliorates major depressive disorder by modulating circadian rhythm: a randomized, double-blind, placebo-controlled trial. J. Pineal Res. 73 (4), e12832. 10.1111/jpi.12832 36073608

[B44] Hirschfeld R. M. (2000). History and evolution of the monoamine hypothesis of depression. J. Clin. Psychiatry. 61 (Suppl. 6), 4–6. 10775017

[B45] Horowitz M. A. Cattaneo A. Cattane N. Lopizzo N. Tojo L. Bakunina N. (2020). Glucocorticoids prime the inflammatory response of human hippocampal cells through up-regulation of inflammatory pathways. Brain. Behav. Immun. 87, 777–794. 10.1016/j.bbi.2020.03.012 32194233

[B46] Hu X. L. Wu X. (2023). Review of traditional Chinese medicines in ameliorating neuropsychiatric diseases by improving the levels of monoamine neurotransmitters via gut microbiota regulation. China J. Chin. Mater. Med. 48 (04), 853–860. 10.19540/j.cnki.cjcmm.20221103.601 36872256

[B47] Huang D. X. Zhao Z. H. Xiao Y. X. He Y. Q. (2023). Berberine improves liver injuries and intestinal flora disorders in high—fat diet—induced non—alcoholic fatty liver disease in rats. J. Clin. Hepatol. 26 (01), 23–26. 10.3969/j.issn.1672-5069.2023.01.007

[B48] Huang Y. Wu J. Zhang H. Li Y. Wen L. Tan X. (2023). The gut microbiome modulates the transformation of microglial subtypes. Mol. Psychiatry. 28, 1611–1621. 10.1038/s41380-023-02017-y 36914812 PMC10208978

[B49] Ida T. Hara M. Nakamura Y. Kozaki S. Tsunoda S. Ihara H. (2008). Cytokine-induced enhancement of calcium-dependent glutamate release from astrocytes mediated by nitric oxide. Neurosci. Lett. 432 (3), 232–236. 10.1016/j.neulet.2007.12.047 18255223

[B50] Joseph J. J. Golden S. H. (2017). Cortisol dysregulation: the bidirectional link between stress, depression, and type 2 diabetes mellitus. Ann. N. Y. Acad. Sci. 1391 (1), 20–34. 10.1111/nyas.13217 27750377 PMC5334212

[B51] Junjie X. Jia L. Jiaoya X. Xinchun Z. (2024). Historical evolution and research progress of the classical prescription of Baihe Dihuang decoction. Jilin. J. Chin. Med. 44 (04), 475–480. 10.13463/j.cnki.jlzyy.2024.04.024

[B52] Juruena M. F. Bocharova M. Agustini B. Young A. H. (2018). Atypical depression and non-atypical depression: is HPA axis function a biomarker? A systematic review. J. Affect. Disord. 233, 45–67. 10.1016/j.jad.2017.09.052 29150144

[B53] Kim I. B. Park S. C. Kim Y. K. (2023). Microbiota-gut-brain Axis in major depression: a new therapeutic approach. Adv. Exp. Med. Biol. 1411, 209–224. 10.1007/978-981-19-7376-5_10 36949312

[B54] Kohler O. Krogh J. Mors O. Benros M. E. (2016). Inflammation in depression and the potential for anti-inflammatory treatment. Curr. Neuropharmacol. 14 (7), 732–742. 10.2174/1570159x14666151208113700 27640518 PMC5050394

[B55] Kovich H. Kim W. Quaste A. M. (2023). Pharmacologic treatment of depression. Am. Fam. Physician 107 (2), 173–181. 36791444

[B56] Kwon H. S. Koh S. H. (2020). Neuroinflammation in neurodegenerative disorders: the roles of microglia and astrocytes. Transl. Neurodegener. 9 (1), 42. 10.1186/s40035-020-00221-2 33239064 PMC7689983

[B57] Lach G. Schellekens H. Dinan T. G. Cryan J. F. (2018). Anxiety, depression, and the microbiome: a role for gut peptides. Neurotherapeutics 15 (1), 36–59. 10.1007/s13311-017-0585-0 29134359 PMC5794698

[B58] Lee B. H. Kim H. Park S. H. Kim Y. K. (2007). Decreased plasma BDNF level in depressive patients. J. Affect. Disord. 101 (1-3), 239–244. 10.1016/j.jad.2006.11.005 17173978

[B59] Li M. Jiang H. Hao Y. Du K. Du H. Ma C. (2022). A systematic review on botany, processing, application, phytochemistry and pharmacological action of Radix Rehmnniae. J. Ethnopharmacol. 285, 114820. 10.1016/j.jep.2021.114820 34767834

[B60] Li S. Zhang G. Zhu R. Fan J. Yang S. (2024). Changes of serum BDNF and TGF-β1 levels in patients with depression and their relationship with the severity of the disease and cognitive function. Shandong Med. J. 64 (16), 77–79. 10.3969/j.issn.1002-266X.2024.16.018

[B61] Li S. X. Han Y. Xu L. Z. Yuan K. Zhang R. X. Sun C. Y. (2018). Uncoupling DAPK1 from NMDA receptor GluN2B subunit exerts rapid antidepressant-like effects. Mol. Psychiatry. 23 (3), 597–608. 10.1038/mp.2017.85 28439098 PMC5822462

[B62] Li Y. (2011). Molecular structure and pharmacological activity of aucubin and its derivatives. Northwestern University.

[B63] Lima G. B. Doorduin J. Klein H. C. Dierckx R. Bromberg E. de Vries E. (2019). Brain-derived neurotrophic factor in brain disorders: focus on neuroinflammation. Mol. Neurobiol. 56 (5), 3295–3312. 10.1007/s12035-018-1283-6 30117106 PMC6476855

[B64] Lindqvist D. Dhabhar F. S. James S. J. Hough C. M. Jain F. A. Bersani F. S. (2017). Oxidative stress, inflammation and treatment response in major depression. Psychoneuroendocrinology 76, 197–205. 10.1016/j.psyneuen.2016.11.031 27960139 PMC5272818

[B65] Liu C. Ma R. Wang L. Zhu R. Liu H. Guo Y. (2017). Rehmanniae Radix in osteoporosis: a review of traditional Chinese medicinal uses, phytochemistry, pharmacokinetics and pharmacology. J. Ethnopharmacol. 198, 351–362. 10.1016/j.jep.2017.01.021 28111216

[B66] Liu F. He Q. Wang H. L. Huang D. Yang W. J. (2021). Chlorogenic acid relieves neurological injury and activation of NLRP3 inflammasomes in Aβ-induced Alzheimer's disease mice. Chin. J. Immunol. 37 (16), 1933–1937. 10.3969/j.issn.1000-484X.2021.16.003

[B67] Liu J. Li Q. Guo L. Li G. Zhao X. Liu J. (2017). Study on acute toxicity and sub-chronic toxicity of the extract of Rehmannia glutinosa. China Anim. Husb. Vet. Med. 44 (11), 3372–3378. 10.16431/j.cnki.1671-7236.2017.11.037

[B68] Liu J. Ren J. Lan J. Gong Y. Liu J. (2024). Research Progresson the Pharmacological Effects of Colchicin. Asia-pacific. Tradit. Med., 1–7.

[B69] Liu J. Wang H. Y. (2015). Function and signal pathways of brain-derived neurotrophic factor in depression. China Med. Her 12 (36), 49–52.

[B70] Liu J. Yang L. Dong Y. Zhang B. Ma X. (2018). Echinacoside, an inestimable natural product in treatment of neurological and other disorders. Molecules 23 (5), 1213. 10.3390/molecules23051213 29783690 PMC6100060

[B71] Liu X. Y. Zhang X. Lv X. Dou W. H. (2023). Antidepressive mechanism of Xiaoyao powder:A study based on network pharmacology and molecular docking. Hunan. J. Tradit. Chin. Med. 39 (03), 148–157+190 10.16808/j.cnki.issn1003-7705.2023.03.034

[B72] Liu Y. Lan N. Liu L. Guo L. S. Fu X. B. Cui W. (2013). Effect of luteolin on depression induced by chronic unpredictable mild stress in mice. Lishizhen Med. Mater. Med. Res. 24 (06), 1382–1384. 10.3969/j.issn.1008-0805.2013.06.040

[B73] Lok A. Mocking R. J. Ruhe H. G. Visser I. Koeter M. W. Assies J. (2012). Longitudinal hypothalamic-pituitary-adrenal axis trait and state effects in recurrent depression. Psychoneuroendocrinology 37 (7), 892–902. 10.1016/j.psyneuen.2011.10.005 22094110

[B74] Lu R. R. Wang H. H. Zhang L. Li M. Lei X. Y. Deng X. Y. (2021). Effects of pyrethrin in Rehmannia glutamate on oxidative stress and NMDAR1 expression in PC-12 cells induced by glutamate. Pharmacol. Clin. Chin. Mater. Med. 37 (05), 45–48. 10.13412/j.cnki.zyyl.2021.05.008

[B75] Lu R. R. Zhang L. Wang H. H. Li M. Deng X. K. Feng W. S. (2022). Study on the inhibitory effect of aucubin on glutamate-induced excitatory neuroto. Chin. J. Mod. Appl. Pharm. 39 (24), 3197–3203. 10.13748/j.cnki.issn1007-7693.2022.24.002

[B76] Lu Z. L. (1997). Compendium on epidemic febrile diseases. Shenyang: Liaoning Science and Technology Press.

[B77] Luo L. M. Pei G. Tan L. Zhou X. J. Zhan J. H. Liao N. (2017). Research progress on chemical constituents and pharmacological effects of medicinal Lilium plants. Tradit. Chin. Drug Res. Clin. Pharmacol. 28 (06), 824–837. 10.19378/j.issn.1003-9783.2017.06.022

[B78] Ma K. Zhang H. X. Dong Z. F. Wei S. Zheng W. J. Wang X. (2019). Research progress of Baihe Dihuang decoction in the treatment of depression. Chin. Tradit. Pat. Med. 41 (04), 874–878. 10.3969/j.issn.1001-1528.2019.04.032

[B79] Mahmoud S. Gharagozloo M. Simard C. Gris D. (2019). NLRX1 enhances glutamate uptake and inhibits glutamate release by astrocytes. Cells 8 (2), 400. 10.3390/cells8050400 31052241 PMC6562695

[B80] Mao Q. Ma K. Wang J. Fang T. Liu W. Zhang H. (2024). Study of the changes of chemical composition in single frying and Co-decoction of Baihe Dihuang decoction based on LC-MS method. J. Liaoning Univ. Tradit. Chin. Med. 26 (02), 77–82. 10.13194/j.issn.1673-842x.2024.02.013

[B81] Mehta V. Parashar A. Udayabanu M. (2017). Quercetin prevents chronic unpredictable stress induced behavioral dysfunction in mice by alleviating hippocampal oxidative and inflammatory stress. Physiol. Behav. 171, 69–78. 10.1016/j.physbeh.2017.01.006 28069457

[B82] Mikulska J. Juszczyk G. Gawronska-Grzywacz M. Herbet M. (2021). HPA Axis in the pathomechanism of depression and schizophrenia: new therapeutic strategies based on its participation. Brain Sci. 11 (10), 1298. 10.3390/brainsci11101298 34679364 PMC8533829

[B83] Mosiolek A. Pieta A. Jakima S. Zborowska N. Mosiolek J. Szulc A. (2021). Effects of antidepressant treatment on peripheral biomarkers in patients with major depressive disorder (MDD). J. Clin. Med. 10 (8), 1706. 10.3390/jcm10081706 33920992 PMC8071355

[B84] Mullins M. E. Robertson D. G. Norton R. L. (2000). Troponin I as a marker of cardiac toxicity in acute colchicine overdose. Am. J. Emerg. Med. 18 (6), 743–744. 10.1016/s0735-6757(00)90317-6 11043640

[B85] Murrough J. W. Abdallah C. G. Mathew S. J. (2017). Targeting glutamate signalling in depression: progress and prospects. Nat. Rev. Drug Discov. 16 (7), 472–486. 10.1038/nrd.2017.16 28303025

[B86] Neupane S. P. Virtej A. Myhren L. E. Bull V. H. (2022). Biomarkers common for inflammatory periodontal disease and depression: a systematic review. Brain Behav. Immun. Health 21, 100450. 10.1016/j.bbih.2022.100450 35330865 PMC8938251

[B87] Olivenza R. Moro M. A. Lizasoain I. Lorenzo P. Fernandez A. P. Rodrigo J. (2000). Chronic stress induces the expression of inducible nitric oxide synthase in rat brain cortex. J. Neurochem. 74 (2), 785–791. 10.1046/j.1471-4159.2000.740785.x 10646531

[B88] Pan G. Xie Z. Huang S. Tai Y. Cai Q. Jiang W. (2017). Immune-enhancing effects of polysaccharides extracted from Lilium lancifolium Thunb. Int. Immunopharmacol. 52, 119–126. 10.1016/j.intimp.2017.08.030 28898768

[B89] Pan W. Chi X. Wang Y. Ma M. Fan J. Xue X. (2023). Ameliorating effect of Baihe Dihuang Decoction on the symptoms of lily disease in rats with Yin deficiency internal heat depression. Chin. Tradit. Pat. Med. 45 (05), 1652–1657. 10.3969/j.issn.1001-1528.2023.05.046

[B90] Pandey G. N. Rizavi H. S. Zhang H. Bhaumik R. Ren X. (2018). Abnormal protein and mRNA expression of inflammatory cytokines in the prefrontal cortex of depressed individuals who died by suicide. J. Psychiatry. Neurosci. 43 (6), 376–385. 10.1503/jpn.170192 30371993 PMC6203549

[B91] Peng W. H. Lo K. L. Lee Y. H. Hung T. H. Lin Y. C. (2007). Berberine produces antidepressant-like effects in the forced swim test and in the tail suspension test in mice. Life Sci. 81 (11), 933–938. 10.1016/j.lfs.2007.08.003 17804020

[B92] Perez-Caballero L. Torres-Sanchez S. Romero-Lopez-Alberca C. Gonzalez-Saiz F. Mico J. A. Berrocoso E. (2019). Monoaminergic system and depression. Cell. Tissue. Res. 377 (1), 107–113. 10.1007/s00441-018-2978-8 30627806

[B93] Plotsky P. M. Owens M. J. Nemeroff C. B. (1998). Psychoneuroendocrinology of depression. Hypothalamic-pituitary-adrenal axis. Hypothalamic-pituitary-adrenal Axis. Psychiatr. Clin. North Amer. 21 (2), 293–307. 10.1016/s0193-953x(05)70006-x 9670227

[B94] Qin Y. Zhang Y. (2020). Research progress on the influence mechanism of kynurenine metabolic pathway on depression. Chin. Pharmacol. Bull. 36 (12), 1640–1644. 10.3969/j.issn.001-1978.2020.12.003

[B95] Qing G. Y. Qu Y. Zhang H. Si G. M. (2023). Research progress on pharmacological action of Baihe Dihuang decoction. Shandong J. Tradit. Chin. Med. 42 (03), 299–303. 10.16295/j.cnki.0257-358x.2023.03.020

[B96] Qu Y. F. (2021). Study on intestinal microecological mechanism of kaempferol in relieving ulcerative colitis in mice. Neimenggu. Med. Univ. 83 10.27231/d.cnki.gnmyc.2021.000370

[B97] Redza-Dutordoir M. Averill-Bates D. A. (2016). Activation of apoptosis signalling pathways by reactive oxygen species. Biochim. Biophys. Acta 1863 (12), 2977–2992. 10.1016/j.bbamcr.2016.09.012 27646922

[B98] Ren L. Chen G. (2017). Rapid antidepressant effects of Yueju: a new look at the function and mechanism of an old herbal medicine. J. Ethnopharmacol. 203, 226–232. 10.1016/j.jep.2017.03.042 28347831

[B99] Sarno E. Moeser A. J. Robison A. J. (2021). Neuroimmunology of depression. Adv. Pharmacol. 91, 259–292. 10.1016/bs.apha.2021.03.004 34099111 PMC8877598

[B100] Shi A. Li T. Zheng Y. Song Y. Wang H. Wang N. (2021). Chlorogenic acid improves NAFLD by regulating gut microbiota and GLP-1. Front. Pharmacol. 12, 693048. 10.3389/fphar.2021.693048 34276380 PMC8278021

[B101] Sies H. (2015). Oxidative stress: a concept in redox biology and medicine. Redox Biol. 4, 180–183. 10.1016/j.redox.2015.01.002 25588755 PMC4309861

[B102] Sim W. S. Choi S. I. Jung T. D. Cho B. Y. Choi S. H. Park S. M. (2020). Antioxidant and anti-inflammatory effects of Lilium lancifolium bulbs extract. J. Food Biochem. 44 (5), e13176. 10.1111/jfbc.13176 32173873

[B103] Sipahi H. Mat A. F. Ozhan Y. Aydin A. (2023). The interrelation between oxidative stress, depression and inflammation through the kynurenine pathway. Curr. Top. Med. Chem. 23 (6), 415–425. 10.2174/1568026623666221223111309 36567285

[B104] Sljivo A. Kulenovic A. D. (2023). Fear, anxiety and depression among Bosnia and Herzegovina citizens during the third wave of COVID-19. Iran. J. Psychiatry 18 (1), 1–10. 10.18502/ijps.v18i1.11407 37159641 PMC10163905

[B105] Song J. Gao X. Tian J. S. Qin X. M. Du G. H. Zhou Y. Z. (2017). Modern research on compatibility mechanism of Chinese materia medica pair. Chin. Herb. Med. 48 (21), 4367–4374. 10.7501/j.issn.0253-2670.2017.21.001

[B106] Song L. Wu X. Wang J. Guan Y. Zhang Y. Gong M. (2021). Antidepressant effect of catalpol on corticosterone-induced depressive-like behavior involves the inhibition of HPA axis hyperactivity, central inflammation and oxidative damage probably via dual regulation of NF-κB and Nrf2. Brain Res. Bull. 177, 81–91. 10.1016/j.brainresbull.2021.09.002 34500039

[B107] Stachowicz K. Sowa-Kucma M. (2022). The treatment of depression - searching for new ideas. Front. Pharmacol. 13, 988648. 10.3389/fphar.2022.988648 36278184 PMC9585175

[B108] Sun J. N. Lian X. X. Sun L. L. Liu J. Duan Z. C. Wang Z. L. (2022). Research progress on main compositions and pharmacological actions of lily. Chin. Wild Plant. Resour. 41 (07), 45–50. 10.7501/j.issn.0253-2670.2017.21.001

[B109] Sun Y. X. Wang X. T. (2005). Zhang's medical practitioner. Shanghai: The second military Med Univ Press.

[B110] Tan Y. Li C. Zhou J. Deng F. Liu Y. (2023). Berberine attenuates liver fibrosis by autophagy inhibition triggering apoptosis via the miR-30a-5p/ATG5 axis. Exp. Cell Res. 427 (2), 113600. 10.1016/j.yexcr.2023.113600 37062521

[B111] Tang A. L. Thomas S. J. Larkin T. (2019). Cortisol, oxytocin, and quality of life in major depressive disorder. Qual. Life. Res. 28 (11), 2919–2928. 10.1007/s11136-019-02236-3 31227958

[B112] Tao W. W. Xiao D. Wu H. R. Shen J. L. Huang X. Y. Xue W. D. (2018). Inhibitory effect of monoclonal antibody NCX-2D2 on isoproterenol-induced arrhythmias in adult rat hearts. Chin. Pharmacol. Bull. 34 (09), 1314–1320. 10.3969/j.issn.1001-1978.2018.09.024

[B113] Tian M. Deng D. Liu C. L. (2022). Research progress on the involvement of intestinal microorganisms in the pathogenesis of depression. Clin. J. Med. Off. 50 (06), 658–660. 10.16680/j.1671-3826.2022.06.34

[B114] Vaglio-Garro A. Kozlov A. V. Smirnova Y. D. Weidinger A. (2024). Pathological interplay between inflammation and mitochondria aggravates glutamate toxicity. Int. J. Mol. Sci. 25 (4), 2276. 10.3390/ijms25042276 38396952 PMC10889519

[B115] Vavakova M. Durackova Z. Trebaticka J. (2015). Markers of oxidative stress and neuroprogression in depression disorder. Cell. Longev. 2015, 898393. 10.1155/2015/898393 PMC4453280 26078821

[B116] Wang B. H. Qiao P. Wang W. Song W. Liu C. Wang X. Y. (2021). Effect of albiziae flos and polygalae Radix alone and their combination on depression-like behavior and CREB and NOX2 expression in Hippocampus of chronic unpredictable stress-induced rats. Chin. J. Exp. Tradit. Med. Formulae. 27 (17), 32–39. 10.13422/j.cnki.syfjx.20211604

[B117] Wang C. F. Tian W. G. Chen J. P. Ren T. Gai X. H. Liu Y. (2022). Research progress on antidepressive effect and mechanism of traditional Chinese medicine. Chin. Herb. Med. 53 (09), 2890–2901. 10.7501/j.issn.0253-2670.2022.09.033

[B118] Wang H. H. Lu R. R. Zhang L. Li M. Deng X. K. Feng W. S. (2021). Rehmannia glutinosa glycoside D inhibits neuroinflammation by regulating M1/M2 polarization of microglia. J. Chin. Med. Mater. 44 (11), 2683–2687. 10.13863/j.issn1001-4454.2021.11.034

[B119] Wang J. Chen R. Liu C. Wu X. Zhang Y. (2021). Antidepressant mechanism of catalpol: involvement of the PI3K/Akt/Nrf2/HO-1 signaling pathway in rat hippocampus. Eur. J. Pharmacol. 909, 174396. 10.1016/j.ejphar.2021.174396 34332921

[B120] Wang J. Cui Y. Feng W. Zhang Y. Wang G. Wang X. (2014). Involvement of the central monoaminergic system in the antidepressant-like effect of catalpol in mice. Biosci. Trends. 8 (5), 248–252. 10.5582/bst.2014.01029 25382440

[B121] Wang J. H. Kang B. Hu Y. E. Xia Z. Q. (2008). Protective effect of catalpol on PC12 cells injured by L-glutamate. Chin. Pharmacol. Bull. (09), 1258–1259.

[B122] Wang L. (2013). Effect of Rehmannia glutinosa stachyose on intestinal microflora in diabetic mice. PUMCH.

[B123] Wang P. Xu J. Kong Q. Qin W. Yang T. Fu W. (2023). Research status of acupuncture in treating depression based on NO-related signaling pathways. J. Clin. Acupunct. Moxibustion. 39 (11), 103–107. 10.19917/j.cnki.1005-0779.023226

[B124] Wang R. Song C. Chen Q. Q. Wang N. N. Zhang D. Z. R W. X. (2023). Study on the antioxidant effects of paeonol, chlorogenic acid and Gallic acid on D-galactose-induced aging mice. J. Shanxi. Univ. Tradit. Chin. Med. 46 (02), 95–99. 10.13424/j.cnki.jsctcm.2023.02.015

[B125] Wang S. Q. Su J. Ma S. P. Fu Q. (2020). Research progress of brain-derived neurotrophic factor in depression. Pharmacol. Clin. Res. 28 (04), 279–282. 10.13664/j.cnki.pcr.2020.04.010

[B126] Wang Y. (2014). Extraction technology and anti-depression activity of saponins from Lilium brownii. China Pharm. 25 (07), 602–604. 10.6039/j.issn.1001-0408.2014.07.09

[B127] Wang Y. L. Wu H. R. Zhang S. S. Xiao H. L. Yu J. Ma Y. Y. (2021). Catalpol ameliorates depressive-like behaviors in CUMS mice via oxidative stress-mediated NLRP3 inflammasome and neuroinflammation. Transl. Psychiatry. 11 (1), 353. 10.1038/s41398-021-01468-7 34103482 PMC8187638

[B128] Wang Y. T. Wang X. L. Feng S. T. Chen N. H. Wang Z. Z. Zhang Y. (2021). Novel rapid-acting glutamatergic modulators: targeting the synaptic plasticity in depression. Pharmacol. Res. 171, 105761. 10.1016/j.phrs.2021.105761 34242798

[B129] Wolf S. A. Boddeke H. W. Kettenmann H. (2017). Microglia in physiology and disease. Annu. Rev. Physiol. 79, 619–643. 10.1146/annurev-physiol-022516-034406 27959620

[B130] Wu H. Liu R. Wang J. Li T. Sun Y. Feng X. (2021). Liquid chromatography-mass spectrometry in-depth analysis and *in silico* verification of the potential active ingredients of Baihe Dihuang decoction *in vivo* and *in vitro* . J. Sep. Sci. 44 (21), 3933–3958. 10.1002/jssc.202100434 34473407

[B131] Wu P. (1963). Shennong materia medica sutra. Beijing: People's Health Publishing House.

[B132] Wu S. An S. C. Chen H. B. Li F. (2014). Orbital frontal cortex D1 dopamine receptor modulate glutamate and NMDA receptor in depression induced by chronic unpredictable mild stress. Acta Psychol. 46 (01), 69–78. 10.3724/sp.j.1041.2014.00069

[B133] Wu Y. F. (2023). Study on research progress of antidepressant effect and mechanism of traditional Chinese medicine based on regulation of neurotransmitters. New J. 55 (03), 28–33. 10.13457/j.cnki.jncm.2023.03.005

[B134] Xie J. Wu W. T. Chen J. J. Zhong Q. Wu D. Niu L. (2023). Tryptophan metabolism as bridge between gut microbiota and brain in chronic social defeat stress-induced depression mice. Front. Cell. Infect. Microbiol. 13, 1121445. 10.3389/fcimb.2023.1121445 36909723 PMC9999000

[B135] Xing Z. Chen Y. Chen J. Peng C. Peng F. Li D. (2024a). Metabolomics integrated with mass spectrometry imaging reveals novel action of tetramethylpyrazine in migraine. Food Chem. 460 (Pt 2), 140614. 10.1016/j.foodchem.2024.140614 39089013

[B136] Xing Z. Peng F. Chen Y. Wan F. Peng C. Li D. (2024b). Metabolomic profiling integrated with molecular exploring delineates the action of Ligusticum chuanxiong hort. on migraine. migraine. Phytomedicine. 134, 155977. 10.1016/j.phymed.2024.155977 39208659

[B137] Xing Z. Yang C. Feng Y. He J. Peng C. Li D. (2024c). Understanding aconite's anti-fibrotic effects in cardiac fibrosis. Phytomedicine 122, 155112. 10.1016/j.phymed.2023.155112 37924690

[B138] Xiong X. J. Hu Z. X. Zong S. J. Yang M. Ye J. H. (2022). Study on the regularity of compound prescription of traditional Chinese Medicine in the treatment of Depression based on National Patent. World Chin. Med. 17 (22), 3242–3246. 10.3969/j.issn.1673-7202.2022.22.020

[B139] Xu F. Xie Q. Kuang W. Dong Z. (2023). Interactions between antidepressants and intestinal microbiota. Neurotherapeutics 20, 359–371. 10.1007/s13311-023-01362-8 36881351 PMC10121977

[B140] Xu M. Wang J. Zhang X. Yan T. Wu B. Bi K. (2020). Polysaccharide from Schisandra chinensis acts via LRP-1 to reverse microglia activation through suppression of the NF-κB and MAPK signaling. J. Ethnopharmacol. 256, 112798. 10.1016/j.jep.2020.112798 32251761

[B141] Xu M. Zhai W. Zhang Y. Pan J. Li J. Huang S. (2023). Kaixin Jieyu Granule attenuates neuroinflammation-induced depressive-like behavior through TLR4/PI3K/AKT/FOXO1 pathway: a study of network pharmacology and experimental validation. BMC Complement. Med. Ther. 23 (1), 156. 10.1186/s12906-023-03970-5 37173696 PMC10182664

[B142] Xu Q. Sun Z. C. Long Y. Zhang L. Pan Y. Z. Li Q. (2022). Analyses on antioxidant activitiy in phenolics and composition and metabolism of flavonoids and related compounds in methanol extracts from bulbs of three Lilium species. J. Plant Resour. Environ. 31 (01), 42–52. 10.3969/j.issn.1674-7895.2022.01.06

[B143] Xue H. Y. Lu Y. N. Fang X. M. Xu Y. P. Gao G. Z. Jin L. J. (2012). Neuroprotective properties of aucubin in diabetic rats and diabetic encephalopathy rats. Mol. Biol. Rep. 39 (10), 9311–9318. 10.1007/s11033-012-1730-9 22810648

[B144] Xue X. Pan J. Zhang H. Lu Y. Mao Q. Ma K. (2022). Baihe Dihuang (Lilium Henryi Baker and Rehmannia Glutinosa) decoction attenuates somatostatin interneurons deficits in prefrontal cortex of depression via miRNA-144-3p mediated GABA synthesis and release. J. Ethnopharmacol. 292, 115218. 10.1016/j.jep.2022.115218 35337919

[B145] Xue X. Y. (2021). Study on berberine in improving cognitive function and mechanism of type 2 diabetes mellitu. North. China: Univ. Techno, 61.

[B146] Xue X. Y. Pan J. Shi H. S. Wang Y. Wu J. Gao Z. L. (2022). Analysis on antidepressant mechanism of verbascoside based on RNA-seq technology. Chin. J. Exp. Tradit. Med. Formulae. 28 (14), 148–157. 10.13422/j.cnki.syfjx.20220618

[B147] Yan R. (2022). The levels and clinical significance of serum glutamate and Γ-aminobutyric acid in patients with depression. Int. J. Psychiatry. 49 (04), 609–611. 10.13479/j.cnki.jip.2022.04.043

[B148] Yan X. F. Xie Y. Y. Chen C. Guo K. J. Zhou Z. Y. Wu Y. (2021). Research progress on chemical composition changes and pharmacological action of prepared Rehmannia glutinosa during processing. Lishizhen Med. Mater. Med. Res. 32 (10), 2493–2495. 10.3969/j.issn.1008-0805.2021.10.50

[B149] Yang L. Ao Y. Li Y. Dai B. Li J. Duan W. (2023). Morinda officinalis oligosaccharides mitigate depression-like behaviors in hypertension rats by regulating Mfn2-mediated mitophagy. J. Neuroinflammation 20 (1), 31. 10.1186/s12974-023-02715-y 36765376 PMC9912533

[B150] Yang L. Chen J. Fang R. Y. (2020). The research progress of Oxidative stress indexes in major depressive disorder. J. Clin. Psychiatry. 30 (03), 208–210.

[B151] Yang L. Y. Wang Y. L. Hao L. W. (2022). Research progress of TCM in treating depression. Inf. Tradit. Chin. Med. 39 (07), 86–89. 10.19656/j.cnki.1002-2406.20220716

[B152] Yang Y. Cui Y. Sang K. Dong Y. Ni Z. Ma S. (2018). Ketamine blocks bursting in the lateral habenula to rapidly relieve depression. Nature 554 (7692), 317–322. 10.1038/nature25509 29446381

[B153] Yu L. Wu W. Z. Zhang Y. Q. You X. Zeng Y. (2023). Research overview of HPA Axis in depression. J. Kunming. Med. Univ. 44 (02), 166–171. 10.12259/j.issn.2095-610X.S20230222

[B154] Yuan Z. Y. Li Z. Y. Zhao H. Q. Gao C. Xiao M. W. Jiang X. M. (2021). Effects of different drying methods on the chemical constituents of Lilium lancifolium Thunb. based on UHPLC-MS analysis and antidepressant activity of the main chemical component regaloside A. J. Sep. Sci. 44 (5), 992–1004. 10.1002/jssc.202000969 33352011

[B156] Zaaijer E. R. van Dijk L. de Bruin K. Goudriaan A. E. Lammers L. A. Koeter M. W. (2015). Effect of extended-release naltrexone on striatal dopamine transporter availability, depression and anhedonia in heroin-dependent patients. Psychopharmacologia 232 (14), 2597–2607. 10.1007/s00213-015-3891-4 PMC4480848 25757673

[B157] Zhang H. Xue X. Pan J. Song X. Chang X. Mao Q. (2021). Integrated analysis of the chemical-material basis and molecular mechanisms for the classic herbal formula of Lily Bulb and Rehmannia Decoction in alleviating depression. Chin. Med. 16 (1), 107. 10.1186/s13020-021-00519-x 34674715 PMC8529377

[B158] Zhang J. Narr K. L. Woods R. P. Phillips O. R. Alger J. R. Espinoza R. T. (2013). Glutamate normalization with ECT treatment response in major depression. Mol. Psychiatry. 18 (3), 268–270. 10.1038/mp.2012.46 22565784 PMC3896297

[B159] Zhang J. N. Liu K. X. (2019). Research progress of catalpol. Drug Eval. Res. 42 (08), 1680–1684. 10.7501/j.issn.1674-6376.2019.08.035

[B160] Zhang J. Q. Wu X. H. Feng Y. Xie X. F. Fan Y. H. Yan S. (2016). Salvianolic acid B ameliorates depressive-like behaviors in chronic mild stress-treated mice: involvement of the neuroinflammatory pathway. Acta Pharmacol. Sin. 37 (9), 1141–1153. 10.1038/aps.2016.63 27424655 PMC5022100

[B161] Zhang L. Lu R. R. Wang H. H. Li M. Feng W. S. Deng X. K. (2022). Protective effect and mechanism of rehmannioside D on PC-12 cells injury induced by corticosterone. Chin. Herb. Med. 53 (11), 3385–3393. 10.7501/j.issn.0253-2670.2022.11.014

[B162] Zhang M. Li A. Yang Q. Li J. Wang L. Liu X. (2021). Beneficial effect of alkaloids FromSophora alopecuroides L. On CUMS-induced depression model mice via modulating gut microbiota. Front. Cell. Infect. Microbiol. 11, 665159. 10.3389/fcimb.2021.665159 33954123 PMC8089385

[B163] Zhang R. Yang Y. Deng A. Kang L. Cheng M. Kang C. (2023). Effect of sulfur fumigation on quality and safety of Lilii Bulbus. China J. Chin. Mater. Med. 48 (03), 660–671. 10.19540/j.cnki.cjcmm.20221031.301 36872229

[B164] Zhang X. L. Ma R. P. (2020). Determination of gallic acid in Lilium bulbus by HPLC-UV. Mod. Food (08), 222–225. 10.16736/j.cnki.cn41-1434/ts.2020.08.075

[B165] Zhang Y. Y. (2018). Study on the relationship between depressive symptoms and serum oxidative stress and inflammatory reaction. Hebei. Med. Univ.

[B166] Zhao H. Tang L. Wu B. Meng P. Liu J. Yang H. (2021a). Baihe dihuangtang improves hippocampal neuron damage in anxious depression model rats by inhibiting NLRP3 inflammasome activation. Chin. J. Exp. Tradit. Med. Formulae. 27 (20), 7–14. 10.13422/j.cnki.syfjx.20212037

[B167] Zhao H. Wang Y. Meng P. Han Y. Qiu X. Tang R. (2021b). Study the mechanism of ganbao capsule on regulating the formation of macrophage subtypes to reverse liver fibrosis. Pharmacol. Clin. Chin. Mater. Med. 37 (01), 160–166. 10.13412/j.cnki.zyyl.2021.01.023

[B168] Zhao L. Li D. Chitrakar B. Li C. Zhang N. Zhang S. (2023). Study on Lactiplantibacillus plantarum R6-3 from Sayram Ketteki to prevent chronic unpredictable mild stress-induced depression in mice through the microbiota-gut-brain axis. Food Funct. 14, 3304–3318. 10.1039/d2fo03708d 36938927

[B169] Zhao N. Isguven S. Evans R. Schaer T. P. Hickok N. J. (2023). Berberine disrupts staphylococcal proton motive force to cause potent anti-staphylococcal effects *in vitro* . Biofilm 5, 100117. 10.1016/j.bioflm.2023.100117 37090161 PMC10113750

[B170] Zhao Y. Li H. Fang F. Qin T. Xiao W. Wang Z. (2018). Geniposide improves repeated restraint stress-induced depression-like behavior in mice by ameliorating neuronal apoptosis via regulating GLP-1R/AKT signaling pathway. Neurosci. Lett. 676, 19–26. 10.1016/j.neulet.2018.04.010 29626654

[B171] Zhao Y. T. Zhang Z. L. Li X. J. Li H. S. Zhang Z. Liu C. Q. (2022). Research progress of classic prescription Baihe Dihuang decoction. J. Guangdong. Pharm. Univ. 38 (03), 137–142. 10.16809/j.cnki.2096-3653.2021120607

[B172] Zhao Z. M. Zhao H. Wang Y. L. Shen Y. Wang Z. X. Meng F. L. (2020). Purification of lily polysaccharides and its regulatory effect on intestinal flora disorders in mice. Sci. Technol. Food Ind. 41 (08), 295–300+306. 10.13386/j.issn1002-0306.2020.08.047

[B173] Zheng J. S. (2005). Collected works of materia medica. Beijing: Chin Ancient Book Publishing House.

[B174] Zhou D. Zhang W. Zhai X. Chen D. Han Y. Ji R. (2024). Advances in neurobiological mechanisms of the antidepressant effects of ketamine. Chin. Pharmacol. Bull. (09), 1622–1627. 10.12360/CPB202305083

[B175] Zhou H. Wang X. D. (1993). Evolution of prescriptions of the golden chamber. Beijing: Chin Traditl Med Publishing House.

[B176] Zhou J. An R. Huang X. (2021). Genus Lilium: a review on traditional uses, phytochemistry and pharmacology. J. Ethnopharmacol. 270, 113852. 10.1016/j.jep.2021.113852 33485985

[B177] Zhou X. Jia R. Chen Y. Lin P. Wang Y. (2023). Preliminary exploration of antidepressant mechanisms of Baihe Dihuang decoction. Fujian J. Tradi. Chin. Med. 54 (07), 48–52. 10.13260/j.cnki.jfjtcm.2023.07014

[B178] Zhou Z. Y. Wang Y. Z. Hu Y. R. Xia S. Wang D. Z. Pang J. (2014). Study of Rhizoma Coptidis alkaloids on promoting sleep in mice. Chin. Pharmacol. Bull. 30 (12), 1752–1756. 10.3969/j.issn.1001-1978.2014.12.028

[B179] Zhu M. Lu C. Li W. (2013). Transient exposure to echinacoside is sufficient to activate Trk signaling and protect neuronal cells from rotenone. J. Neurochem. 124 (4), 571–580. 10.1111/jnc.12103 23189969

[B180] Zhu S. H. Xie M. (2022). Progress in pharmacological research of Baihe Dihuang decoction. J. Guangzhou. Univ. Tradit. Chin. Med. 39 (03), 719–726. 10.13359/j.cnki.gzxbtcm.2022.03.042

[B181] Zou D. C. Liu H. S. (1989). General treatise on febrile diseases. Beijing: People's Health Publishing House.

[B182] Zunszain P. A. Anacker C. Cattaneo A. Carvalho L. A. Pariante C. M. (2011). Glucocorticoids, cytokines and brain abnormalities in depression. Prog. Neuro-Psychopharmacol. Biol. Psychiatry. 35 (3), 722–729. 10.1016/j.pnpbp.2010.04.011 PMC3513408 20406665

